# Aldolase A and Phospholipase D1 Synergistically Resist Alkylating Agents and Radiation in Lung Cancer

**DOI:** 10.3389/fonc.2021.811635

**Published:** 2022-01-21

**Authors:** Yu-Chan Chang, Peter Mu-Hsin Chang, Chien-Hsiu Li, Ming-Hsien Chan, Yi-Jang Lee, Ming-Huang Chen, Michael Hsiao

**Affiliations:** ^1^ Department of Biomedical Imaging and Radiological Sciences, National Yang Ming Chiao Tung University, Taipei, Taiwan; ^2^ Department of Oncology, Taipei Veterans General Hospital, Taipei, Taiwan; ^3^ Faculty of Medicine, National Yang Ming Chiao Tung University, Taipei, Taiwan; ^4^ Institute of Biopharmaceutical Sciences, National Yang Ming Chiao Tung University, Taipei, Taiwan; ^5^ Genomics Research Center, Academia Sinica, Taipei, Taiwan; ^6^ Center of Immuno-Oncology, Department of Oncology, Taipei Veterans General Hospital, Taipei, Taiwan; ^7^ Department of Biochemistry, College of Medicine, Kaohsiung Medical University, Kaohsiung, Taiwan

**Keywords:** alkylating agents, radiation, PLD, ALDOA, lung cancer

## Abstract

Exposure to alkylating agents and radiation may cause damage and apoptosis in cancer cells. Meanwhile, this exposure involves resistance and leads to metabolic reprogramming to benefit cancer cells. At present, the detailed mechanism is still unclear. Based on the profiles of several transcriptomes, we found that the activity of phospholipase D (PLD) and the production of specific metabolites are related to these events. Comparing several particular inhibitors, we determined that phospholipase D1 (PLD1) plays a dominant role over other PLD members. Using the existing metabolomics platform, we demonstrated that lysophosphatidylethanolamine (LPE) and lysophosphatidylcholine (LPC) are the most critical metabolites, and are highly dependent on aldolase A (ALDOA). We further demonstrated that ALDOA could modulate total PLD enzyme activity and phosphatidic acid products. Particularly after exposure to alkylating agents and radiation, the proliferation of lung cancer cells, autophagy, and DNA repair capabilities are enhanced. The above phenotypes are closely related to the performance of the ALDOA/PLD1 axis. Moreover, we found that ALDOA inhibited PLD2 activity and enzyme function through direct protein–protein interaction (PPI) with PLD2 to enhance PLD1 and additional carcinogenic features. Most importantly, the combination of ALDOA and PLD1 can be used as an independent prognostic factor and is correlated with several clinical parameters in lung cancer. These findings indicate that, based on the PPI status between ALDOA and PLD2, a combination of radiation and/or alkylating agents with regulating ALDOA-PLD1 may be considered as a new lung cancer treatment option.

## Introduction

Although alkylating agents and radiation exposure have anti-cancer effects, therapeutic resistance is common (1, 2). Previous studies have shown that these approaches can effectively increase cell apoptosis, DNA damage, and proliferation inhibition (3). However, the detailed mechanism of resistance by metabolic reprogramming remains to be investigated. In addition, alkylating agent resistance has similar consequences and characteristics to radiation resistance. Recently, it has been discovered that metabolic reprogramming may be a clue to distinguish between sensitivity and resistance for these anti-cancer therapies (4, 5). Possible reasons include that metabolites are involved in DNA repair, cell apoptosis, autophagy, and cell cycle. Due to the fact that metabolism includes comprehensive intermediates and complex networks. Currently, there is no clear direction or conclusive evidence that this metabolic reprogramming is related to radiation. Therefore, the performance of metabolism-related genes predicted by a large amount of omics data can be compared with the production or consumption of various metabolites as a screening platform to find critical candidates.

PLD is a transphosphatidylase protein, which is mainly hydrolyzed under physiological conditions and can catalyze the conversion of phosphatidylcholine (PC) into choline and phosphatidic acid (PA) (6). PLD is known to be involved in fatty acid synthesis, cytoskeleton dynamics, and secondary messenger transduction (7–9). This protein also plays a role in signaling transduction through interactions with GTPases, phosphatases, and kinases *via* protein–protein interaction (PPI) (10). Moreover, PLD is also essential in regulating cell proliferation and metastasis (11–14). Recently, PLD has been implicated in inhibiting cell apoptosis and has been shown to contribute to cell survival in cancer (15). Elevated PLD activity induces mitogen-activated protein kinase (MAPK) and mammalian target of rapamycin (mTOR), which act as oncogenic signals in cancer (16–18). Increased PLD activity also inhibits tumor suppressor p53 (TP53) and protein phosphatase 2A (PP2) (19–21). Furthermore, modeling suggests that PLD can enhance migration and survival signals in cancer progression (22). Therefore, researchers hypothesize that PLD remains active and promotes the metastasis of stressed cells (23–25). However, the homeostasis of intracellular PLD and the role of each family member remains to be considered.

In lung cancer studies, several metabolic-related enzymes are expressed abnormally in tumorigenesis. Among them, aldolase is one of the candidates. Aldolase relies on its enzymatic activity to promote lactate production and glucose consumption (26), and it binds to other partners through protein–protein interaction (PPI) for further signal transduction (27, 28). Aldolase converts fructose-1,6-biphosphate (F1,6BP) into glyceraldehyde 3-phosphate (G3P) and dihydroxyacetone phosphate (DHAP) (26). The members of the aldolase family are aldolase A, aldolase B, and aldolase C (26). Aldolase A (ALDOA) is ubiquitous in humans. Aldolase B is specifically located in the liver, while aldolase C is expressed in the central nervous system (CNS). Previous reports describe that ALDOA is one of the components that directly interact with PLD2 in the pleckstrin homology (PH) domain and inhibits the enzyme activity of PLD2 (29). In this study, we provide evidence that ALDOA directly binds to PLD2 to suppress its specific enzymatic activity, resulting in an increase in PLD1 levels and an increase in total PLD enzyme activity to promote lung cancer cell invasion. Moreover, we have observed that lung cancer cells can induce ALDOA and PLD after alkylating agents/radiation exposure and acquire various aggressive cancer phenotypes, such as proliferation, DNA repair, and autophagy. These results suggest targeting the ALDOA/PLD axis as a novel therapeutic direction for overcoming the recurrence or progression of locally advanced lung cancer, particularly after chemotherapy/radiotherapy.

## Materials and Methods

### Cell Culture and Stable Clone Establishment

The human lung cell lines H1355, H460, CL1-0, and CL1-5, were grown in RPMI 1640 medium supplemented with 10% Fetal Bovine Serum (FBS) (Invitrogen, Carlsbad, CA, USA). The human lung cell line H1299 was grown in Dulbecco’s Modified Eagle Medium (DMEM) supplemented with 10% FBS (Invitrogen, Carlsbad, CA, USA). The human lung cell line A549 was grown in F12K supplemented with 10% FBS. All cells were incubated under a humidified atmosphere of 5% CO_2_ at 37°C. The lentiviral PLD1/PLD2 shRNA constructs were purchased from the National RNAi Core Facility Platform (Academia Sinica, Taiwan), and the PLD1/PLD2 overexpression plasmids were purchased from Addgene (Watertown, MA, USA) to establish the stable cell lines. Lentiviruses were used to infect the cells for two days. Stable clones were selected with 1 μg/ml puromycin (Sigma, St. Louis, MO, USA) for two weeks. The cell lines CL1-0 and CL1-5 were established and provided as a gift from Dr. Pan-Chyr Yang (National Taiwan University, Taipei, Taiwan). The cell lines A549, H460, H1355, and H1299 were purchased from the American Type Culture Collection (ATCC, Manassas, VA, USA) cell bank.

### Gene Construction and Lentivirus Production

Lentiviral envelope and packing plasmid (pMDG and p△8.91) were purchased from the National RNAi core facility (Academia Sinica, Taiwan). plenti6.3-ALDOA lentiviral constructs and empty vector were purchased from CLONTECH (USA). Lentiviruses were co-transfected into 293T cells with pMDG, p△8.91, and the plasmid construct using a calcium phosphate transfection method. After 48 hours of incubation, lentiviruses were collected and used to infect the cells with polybrene (2μg/ml). The cells with altered ALDOA expression were selected with blasticidin (2 μg/ml) for two weeks.

### Total Phospholipase, Phospholipase D2 ELISA Determination, and Phosphatidic Acid Production Analyses

Total phospholipase D enzyme activity was measured using a colorimetric PLD activity kit (Cat# No. K725, BioVision, Milpitas, CA, USA) and a phospholipase D2 ELISA kit (Cat# No. SED842hu, USCN, Houston, TX, USA), and phosphatidic acid production (Cat# No. KA1383, Abnova, Taoyuan, Taiwan) was according to the manufacturer’s protocols. After an appropriate incubation time, the optical density was then measured at 570 (phospholipase D activity assay)/450 nm (phospholipase D2) wavelengths.

### Western Blot Analysis

Western blot analysis was performed with primary antibodies directed against ALDOA (Cat No. T0891, Epitomics, Cambridge, MA, USA), PLD1 (Cat No. ab189191, Abcam, Cambridge, MA, USA), PLD2 (Cat No. ab78907, Abcam, Cambridge, MA, USA), Ki-67 (Cat No. M7240, Agilent), PCNA (Cat No. 60097-1, Proteintech), PARP (Cat No. ab32138, Abcam), Bax (Cat No. GTX109683, GeneTex), LC3B (Cat No. 18752-1, Proteintech), LAMP2 (Cat No. ab199946, Abcam), RRM2 (Cat No. GTX11044, GeneTex), γ-H2AX (Cat No. A700-053, ThermoFisher) or α-tubulin (Sigma, St. Louis, MO, USA). Immunoreactive bands were visualized using an enhanced chemiluminescence (ECL) system (Amersham ECL Plus™, GE Healthcare Life Sciences, Chalfont St. Giles, UK).

### Chemicals

Cisplatin (Cat. No. ALX-400-040-M050) was purchased from the Enzo Life Sciences; temozolomide (Cat. No. T2577) was purchased from the Sigma-Aldrich; VU0359595 (Cat. No. SML0566) and CAY10594 (Cat. No. F5807) were purchased from the Sigma-Aldrich; MG132 (Cat. No. sc-351846) were purchased from the Santa Cruz Biotechnology. All powders and compounds are dissolved in DMSO solution. Based on previous studies, we have chosen 10μM of cisplatin, 100μM of temozolomide, 10μM of VU0359595, and 50μM of CAY10594 in this study.

### Caspase 3 Activity Assays

Caspase assays were performed on white 96-well plates according to the manufacturer’s protocol using caspase-3 Glo (Promega, USA). Approximately 20,000 cells were seeded onto the 96-well plate, and paclitaxel was added to the cells at 24 h before the caspase assay. The luciferase activity was measured using a Victor3 photometer, and the relative caspase activity was normalized with the corresponding AlamarBlue values.

### Colony Forming Assays

About 1000 cells were evenly mixed and seeded in a 6-well plate and incubated at 37 °C with 5% CO_2_ for two weeks. After that, the culture medium was discarded, cells were washed thrice with PBS, fixed with 4% paraformaldehyde at room temperature for 20 minutes, and stained with crystal violet for 60 minutes. After slowly removing crystal violet, the plates were air-dried and placed under a microscope for counting clones with more than 50 cells. The experiment was conducted three times.

### Cell Viability Assays

Cell viability was determined using the TACS tetrazolium salt 3-(4,5-dimethylthiazol-2-yl)-2, 5-diphenyltetrazolium bromide (MTT) cell proliferation assay kit (Trevigen, Gaithersburg, MD, USA) according to the manufacturer’s instructions. MTT is used to determine cell viability in cell proliferation and cytotoxicity assays. The cells were seeded at a concentration of 2,000 cells/100 μL culture media per well into 96-well microplates. At 24 hours post-seeding, the cells were treated with dimethyl sulfoxide (DMSO) solvent control or different doses of regorafenib for 24, 48, or 72 hours. Subsequently, the cells were incubated in a medium containing MTT for 4 hours, lysed by DMSO, and the optical density at 570 nm was measured using a microplate reader (Spectral Max250; Molecular Devices, Sunnyvale, CA, USA).

### *In Silico* Datasets and Biostatistical Analysis

**Figure 1:**
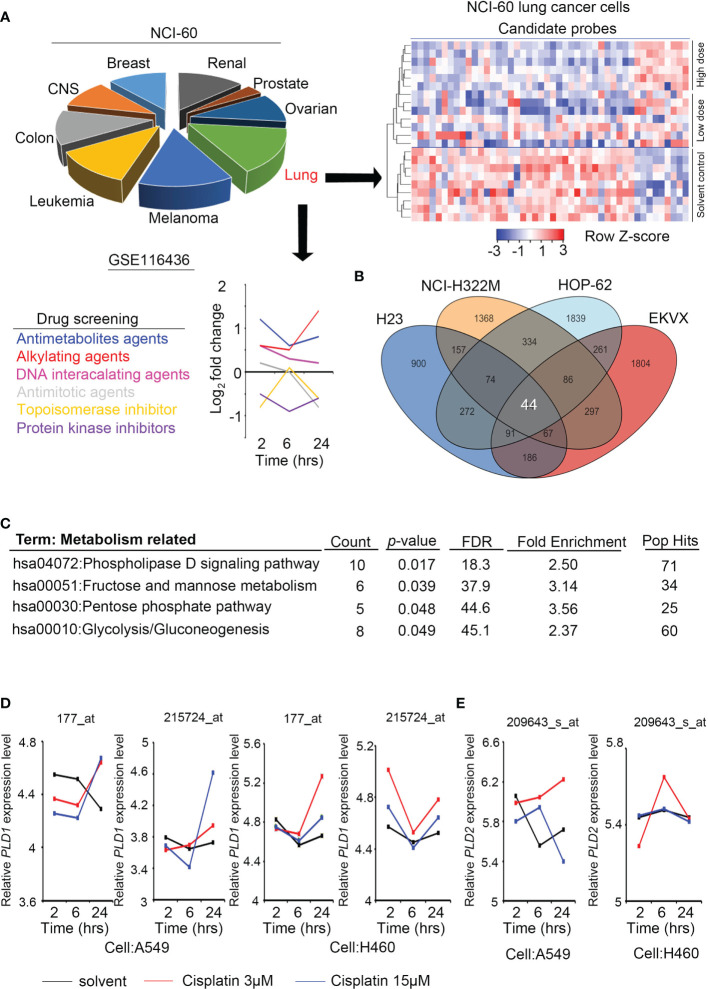
Phospholipase D expression in lung cancer cells with alkylating agent events. **(A)** The cell models in the GSE116436 profile are classified according to NCI-60, and only lung cancer models are extracted and quantified for various drug response analyses. The heat map contains candidates whose high/low dose vs. control >1.5 fold-changes. **(B)** The Venn diagram shows the overlapping targets of GSE116436 during treatment of the alkylating agents in four lung cancer cells (H23, NCI-322M, HOP-62, and EKVX). **(C)** KEGG predicts the highest ranking of metabolic and canonical pathways based on selected features of the 44 candidates in panel **(B)**. **(D)** The expression level of PLD1 in lung cancer cell lines was detected in the GSE116436 profile. Conditions include cisplatin 3 μM and 15 μM. The drug treatments were 2, 6, and 24 hours, respectively. **(E)** The expression level of PLD2 in lung cancer cell lines was detected in the GSE116436 profile. Conditions include cisplatin 3 μM and 15 μM. The drug treatments were 2, 6, and 24 hours, respectively.

**Figure 2:**
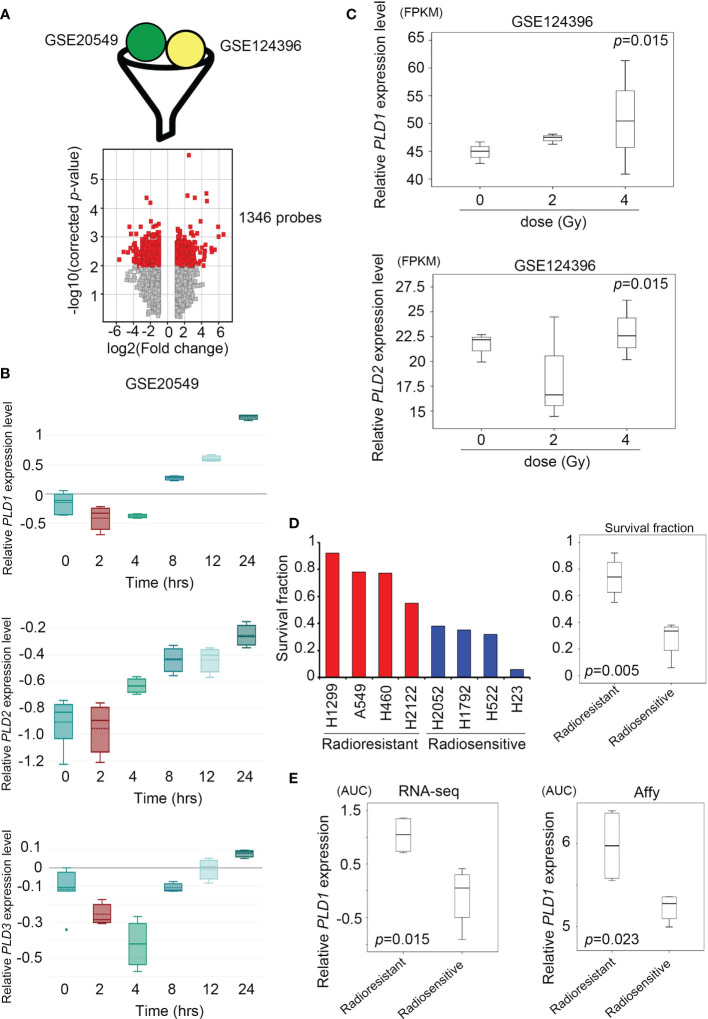
| Lung cancer cells activate the expression of the phospholipase D family under radiation stimulation. **(A)** Combine the GSE20549 and GSE124396 datasets and filter suitable targets. These data are obtained after cut-off and normalization (upper); the volcano plot showed that 1346 probes have become targets in the two datasets and changed to > log2 fold-change (lower). **(B)** Quantify the expression levels of PLD1 (upper), PLD2 (middle), and PLD3 (lower) at each time point (0, 2, 4, 8, 12, and 24 hours) after radiation exposure in the GSE20549 profile. **(C)** Quantify the expression levels of PLD1 and PLD2 at each time point (0, 2, and 4 hours) after radiation exposure in the GSE124396 profile. **(D)** Quantify the survival fraction of various lung cancer cells, and quantify the average of the radioresistant and radiosensitive groups. **(E)** Quantify the expression level of PLD1 in the radioresistant and radiosensitive group (left) microarray probes and (right) RNA-sequencing system. The significance of the difference was analyzed using the nonparametric Mann–Whitney *U* test. *p < 0.05.

For **Figure 1A**, we downloaded GSE116436 from the GEO website. After normalization, we output all probes and selected candidate genes for heatmap analysis. Moreover, we calculated the p-value of each probe in the lung cancer cell lines (n = 8) through the Student’s t-test. In addition, we downloaded GSE20549 and GSE124396 from the GEO website (https://www.ncbi.nlm.nih.gov/geo/) and quantified the PLD1/PLD2 expression level at various conditions in **Figures 2B, C**. Previous studies established metabolomics profiles (30) by collecting CCLE (Cancer Cell Line Encyclopedia, CCLE website (https://portals.broadinstitute.org/ccle/data)) panels and various metabolites (n = 225) to measure cell lines (n = ~928). We chose target events, including LPC and LPE, to classify with our available radioresistance through survival radiation assays (**Figure 4A**). We also collected metabolite-dependency associations from **Supplementary Table 3** for interpretation (**Figure 4B**).

### Clonogenic Survival Assays

The cells were trypsinized and resuspended into T-25 flasks for radiation exposure at different dosages using an X-ray machine. The T-25 flasks were put on ice immediately after radiation exposure. Cells were then seeded into 6 cm dishes with technical triplicates and maintained in a humidified incubator without disturbance. After seven days of incubation, the 6 cm dishes were collected, washed gently with 1X phosphate-buffered saline (PBS), and stained with 0.02% crystal violet for 10 min. The dishes were further rinsed and subjected to microscopic examination to quantify colony number (each colony contained more than 50 cells). The plating efficiency was determined as the ratio of the number of colonies divided by seeded cells. The surviving fraction was determined from the ratio of plating efficiency of the irradiated cells compared to that of the unirradiated controls.

### Radiation

Using a RS 2000 X-ray Biological Irradiator (RS 2000; Rad Source Technologies, Suwanee, GA, USA), some exponentially growing breast and lung cancer cell lines were exposed to various doses of radiation (0–10 Gy). The dose rate was 1.03 Gy/min, and the source-to-bolus distance was 80 cm.

### Immunoprecipitation Analysis

Whole-cell lysates (2 mg) from culture cells were incubated overnight with corresponding antibodies ALDOA (Cat No. T0891, Epitomics, Cambridge, MA, USA), PLD1 (Cat No. ab189191, Abcam, Cambridge, MA, USA), PLD2 (Cat No. ab78907, Abcam, Cambridge, MA, USA). Antibodies-interacting proteins were purified according to the manufacturer’s protocol.

### Clinical Lung Cancer Patient Cohort

In total, 107 patients diagnosed with non-small cell lung cancer at the Kaohsiung Medical University Hospital in Taiwan from 1991 to 2007 were included in this study. According to the seventh edition of the Cancer Staging Manual of the American Joint Committee on Cancer (AJCC), all cases were staged. The histological cancer type was classified according to the World Health Organization (WHO) 2004 classification. Follow-up data were available in all cases, and the longest clinical follow-up time was 190 months. Clinical information and pathology data were collected *via* a retrospective review of patient medical records. Overall survival and disease-free survival were defined as the intervals from surgery to death caused by non-small cell lung cancer and recurrence, or distant metastasis. The study was performed with the approval and permission of the Institutional Review Board (KMUH-IRB-2011-0286).

### Immunohistochemical Staining

Each tumor sample was obtained from the formalin-fixed paraffin-embedded tissues and selected and exhibited morphology typical of the diagnosis. Assessable cores were obtained in 107 cases. The histopathological diagnoses of all samples were reviewed and confirmed by two pathologists, Chia-Yi Su and Michael Hsiao. Serial 5-μm thick tissue sections cut from the tissue microarray were performed with immunohistochemistry staining by using an automated immunostainer (Ventana, Tucson, AZ, USA). In brief, the tissue sections were dewaxed, deparaffinized, and rehydrated by 60°C ovens, xylene, and gradient alcohol. Incubation in Tris-EDTA buffer for antigen retrieval. Polyclonal rabbit anti-human ALDOA antibody (1:100, Epitomics, Cambridge, MA, USA), anti-human PLD1 and PLD2 antibodies (1:100, GeneTex, Hsinchu, Taiwan) was used to stain slides. The scoring of IHC staining intensity was as follows: less than 10% of cytoplasmic staining, including no staining, in the tumor cells, was defined as score 0, more than 10% barely partial cytoplasmic staining in the tumor sections was defined as score 1+, more than 10% moderate cytoplasmic staining of tumor cells was defined as score 2+, and intense cytoplasmic staining in >10% of the tumor cells was defined as score 3+. Scoring 0 and 1+ indicated low protein 1 expression, whereas 2 scores of 2+ and 3+ were defined as high expression.

### Statistical Analysis

Non-parametric Mann-Whitney *U*-test was utilized to evaluate the statistical significance of the data from independent experiments. Statistical analyses were performed by SPSS 17.0 software (Statistical Package for the Social Sciences, Chicago, IL, USA). We used the paired *t*-test to compare the ALDOA/PLD1/PLD2 expression levels in cancer tissues and corresponding normal adjacent tissues. Pearson’s chi-square test analyzed the correlations between the ALDOA/PLD1/PLD2 IHC expression levels and clinicopathological variables. The survival rates were calculated using the Kaplan-Meier method and comparison by using the log-rank test. When the patient was lost during follow-up, the follow-up time was censored. Multivariate and univariate analyses were estimated through Cox proportional hazards regression analysis with and without adjusting for ALDOA/PLD1/PLD2 IHC expression level, tumor stage, lymph node stage, and metastasis. A *P*-value of <0.05 was considered significant in all analyses.

## Results

### Phospholipase D Participates in the Response of Lung Cancer Cells to Alkylating Agent Exposure

To analyze the response of cancer cells after treatment with alkylating agents, we collected and re-analyzed the existing public databases in the NCI-60 project (GSE116436). This dataset contains many types of used drugs, including antimetabolites, alkylating agents, DNA-interacting agents, antimitotic, topoisomerase inhibitors, and protein kinase inhibitors. We analyzed the data and selected the groups that had a consistent trend from low doses of drugs to solvents and from high doses of drugs to solvents. In addition, compared with the short-term group, we expected to observe a significant cumulative effect in the long-term group. Most of the groups that met the above criteria were the alkylating agent groups, and regarding NCI-60 classification, the lung cancer cell response was the most significant (**Figure 1A**). To explore such findings, we further overlapped four lung cancer cell lines (H23, NCI-H322M, HOP-62, and EKVX). A total of 44 probes meet the standard and had similar trends (**Figure 1B**).

We predicted common signatures through the KEGG and DAVID website tools, and the results revealed that specific metabolic processes and phospholipase D pathways were proposed. The phospholipase D family member PLD1/PLD2 was selected for further study based on the predicted rankings and statistical values (**Figure 1C**). We examined the expression levels of PLD1 and PLD2 in some lung cancer cell lines (A549 and H460) of the same cohort (GSE116436). The results showed that PLD1 has a clear trend in performance, while PLD2 does not (**Figures 1D, E**). Based on this information, we speculate that the PLD family, particularly PLD1, participates in the response to alkylating agent treatment.

### High Expression of Phospholipase D in Radiation Model of Lung Cancer Cells

To distinguish whether radiation exposure induces events similar to alkylating agents in lung cancer cells, we recruited two independent experiments based on array chips, including GSE20549 and GSE124396, which contained multiple radiation doses or time courses for H460, A549, and H1299 cells. In previous studies, H460 was considered to be a radiosensitive cells, while H1299 and A549 were radioresistant (31, 32). We merged the typical signature of H1299 and A549, then excluded the similarity with H460. We obtained a list of 1346 probes for further interpretation (**Figure 2A**). At the same time, we identified the expression levels of all family members (PLD1~PLD6) and observed that PLD1 is unique and cumulative after radiation in these datasets (GSE20549) (**Figure 2B** and **Supplementary Figure S1**). On the other hand, we calculated the expression of PLD1 and PLD2 in different doses of radiotherapy (GSE124396). The data indicate that PLD1 overexpression is correlated with radiation dose (**Figure 2C**). In addition to these cohorts, we also recruited lung cancer gene expression and survival rates after irradiation in previous studies (33). This study confirmed the radiation response of 70 lung cancer cell lines using the same dose and measurement method (high survival fraction means radioresistance, and vice versa). We divided these lung cancer cell lines into radioresistant and radiosensitive groups (**Figure 2D**). Furthermore, we detected that PLD1 mRNA was induced in the radioresistant group compared with the radiosensitive group (**Figure 2E**). In contrast, PLD2 mRNA expression did not change significantly. These results indicated that alkylating agents or radiation exposure in lung cancer cells could induce PLD1 expression.

### Phospholipase D1 Inhibitor Modulates Various Characteristics of Lung Cancer Cells After Adding Alkylating Agents

**Figure 3:**
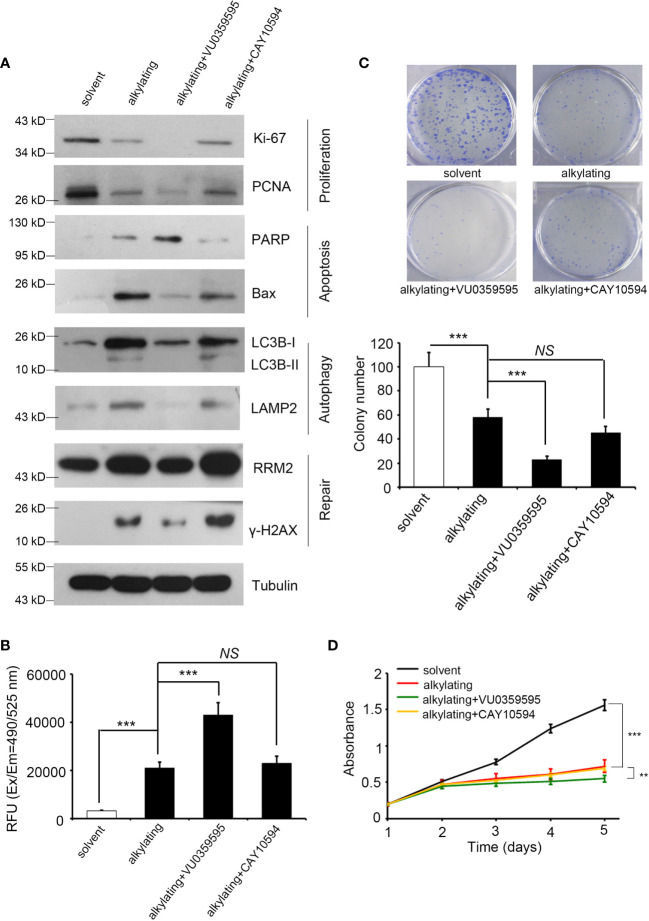
Inhibited PLD1 expression induces apoptosis and attenuates proliferation, autophagy, and repair by alkylating agent treatment. **(A)** The protein levels of Ki-67, PCNA, PARP, BAX, LC3B, LAMP2, γ-H2AX, and RRM2 in A549 cells after alkylating agent exposure, with or without specific inhibitor treatment (PLD1-specific, VU0359595, 10 μM; and PLD2-specific, CAY10594, 50 μM). Tubulin serves as an internal control. **(B)** Quantitation of the activity of Caspase 3 in A549 cells after alkylating agent exposure with or without specific inhibitors treatment (PLD1-specific, VU0359595, 10 μM; and PLD2-specific, CAY10594, 50 μM). **(C)** Quantitation of colony-forming ability in A549 cells after alkylating agent exposure with or without specific inhibitors treatment (PLD1-specific, VU0359595, 10 μM; and PLD2-specific, CAY10594, 50 μM). **(D)** A measure of the growth curve in A549 cells after alkylating agent exposure with or without specific inhibitor treatment (PLD1-specific, VU0359595; and PLD2-specific, CAY10594). In this study, 10 μM of cisplatin, 100 μM of temozolomide, 10 μM of VU0359595, and 50 μM of CAY10594. The data from three independent experiments are presented in **(B–D)** as the means ± SEM. The significance of the difference was analyzed using the nonparametric Mann–Whitney *U* test. **p < 0.01, ***p < 0.001. ns, non significant.

To prove that the consequences of radiation and alkylating agent incidents are indeed affected by PLD, we recruited two specific inhibitors to block PLD1 (VU0359595) and PLD2 (CAY10594) for further experiments, respectively. These PLD inhibitors are designed through isomeric structures and have PLD selectivity (34, 35). We first treated the lung cancer cell line A549 with alkylating drugs (cisplatin or temozolomide) to simulate the previous in silico dataset, then added PLD1/PLD2 inhibitors, respectively. We subsequently examined the various cancer cell events that can be regulated, including cell proliferation, apoptosis, autophagy, and DNA repair (**Figure 3A**). When treated with alkylating agents or radiation, cancer cells are destroyed and die. In addition to slowing down the efficiency of proliferation, this treatment will also initiate apoptosis and autophagy, as well as DNA repair mechanisms. We selected Ki-67 and proliferating cell nuclear antigen (PCNA), the participants in DNA replication. Bax and PARP have been proven to be intrinsic pathways to induce apoptosis, and LC3B and LAMP2 can maintain lysosomal stability and are involved in autophagy. RRM2 and γ-H2AX are responsible for DNA synthesis and repair. We observed that apoptosis, autophagy, and DNA repair markers were upregulated, and the proliferation protein Ki-67/PCNA was inhibited in the alkylating agents group. Compared with the alkylating agent alone, the combined use of VU0359595 and alkylating agents can more significantly inhibit autophagy and DNA repair ability, while increasing the anti-proliferation and apoptosis rate (**Figure 3A**), having a stronger toxic effect on cancer cells. On the other hand, CAY10594 is a specific inhibitor of PLD2. Compared with the alkylating agent group, although its proliferation ability was slightly reduced, CAY10594 did not increase cell apoptosis, and it also enhanced DNA repair ability (**Figure 3A**). In addition, we observed that the combination of alkylating agents and VU0359595 also affects the activity of caspase-3 and the colony-forming ability of lung cancer cells (**Figures 3B, C**). However, there was still not a great difference in the PLD2 inhibition group (**Figures 3B, C**). Our results also provide evidence that the additional input of VU0359595 and CAY10594 does not cause potent cytotoxicity (**Figure 3D**). Although the combination of alkylating agents and VU0359595 induced cell apoptosis signaling and reduced proliferation, it had no significant effect on cell viability. Our assessment is that it may have caused cell cycle arrest in our experiments, but more evidence is needed. This phenomenon indicates that PLD1 dominates the apoptosis and phenotypic series.

### PLD1 and Its Metabolites Rely on ALDOA-Based Protein-Protein Interactions

**Figure 4:**
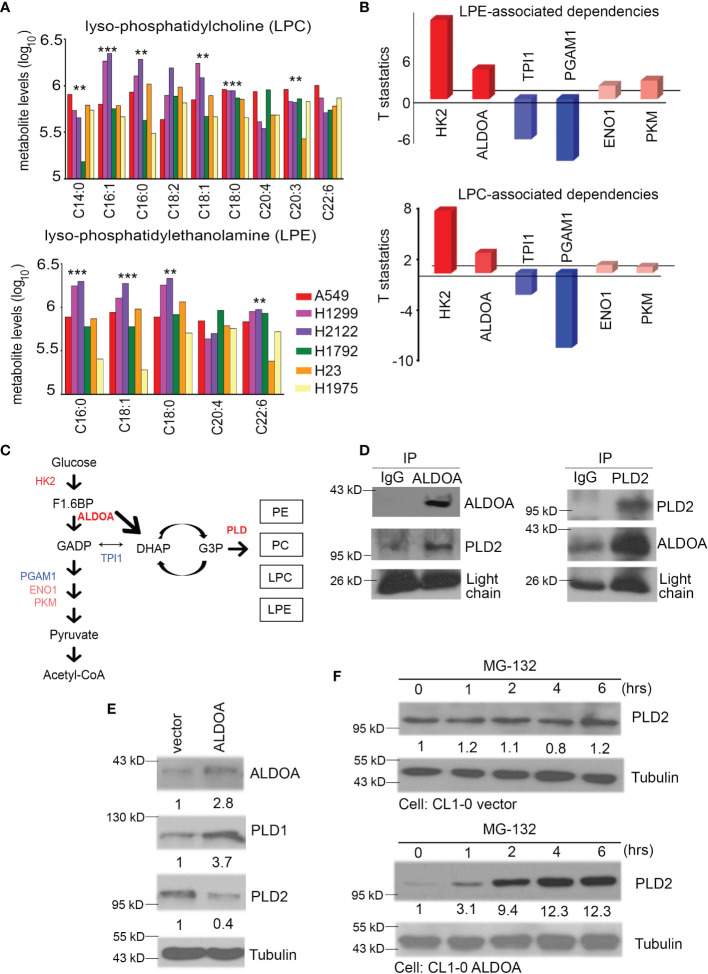
Events and activities related to PLD depend on ALDOA. **(A)** The production of lysophosphatidylcholine (LPC) and lysophosphatidylethanolamine (LPE) were obtained from the available CCLE metabolomics results. Combined with previous research, we collected the survival fraction after radiation exposure in each CCLE cell. A549, H1299, H2122 (radioresistant group), and H1792, H23, H1975 (radiosensitivity group) lung cancer cells were divided into two groups to quantify their LPC and LPE concentrations. **(B)** The dependence of LPC or LPE products on glycolytic pathways from the available CCLE metabolites profile. These configuration files are calculated based on the RNA-seq expression of each candidate in the CCLE omics database. **(C)** Trends between glycolysis and phospholipase D metabolic pathways. This result shows that the performance of LPC/LPE depends on HK2, ALDOA, and PLD. The importance of TPI1 has decreased. Red color means positive dependence. Blue color means negative dependence for LPC/LPE products. **(D)** Two-way model of immunoprecipitation using ALDOA and PLD2 antibodies in CL1-0 cells with or without forced expression of an exogenous ALDOA gene. IgG served as the negative control. **(E)** The protein levels of ALDOA, PLD1, and PLD2 in A549 cells with or without shALDOA. Tubulin serves as an internal control. **(F)** The PLD2 protein level after treatment with MG-132 is time-dependent in A549 cells with vector control or ALDOA overexpression. Tubulin serves as an internal control. The significance of the difference was analyzed using the nonparametric Mann–Whitney *U* test.

We dissected the levels of several metabolites regulated by PLD and found that the products of lysophosphatidylcholine (LPC) and lysophosphatidylethanolamine (LPE) of radiation-resistant cell lines A549, H1299, and H2122 were higher than those of the radiation-sensitive cell lines H1792, H23, and H1975 from previous studies (30) (**Figure 4A**). According to the previous theories, the heterogenicity of glycolysis in cancer may be a therapeutic opportunity as it affects the drug response, tumor microenvironment, and drug flux (including alkylating agents) (36). We speculated that glycolysis and its branches pentose phosphate pathway (PPP) and fructose metabolism may reflect changes in lung cancer after the addition of alkylating agents (**Figure 1C**). We further identified some genes related to glycolysis from the CCLE and summarized their dependence on each metabolite. Our results indicated that HK2 and ALDOA have more positive correlation effects than other enzymes (**Figure 4B**). In particular, ALDOA accelerates the carboxylate metabolism involved in the shuttle of DHAP-G3P, thereby linking lipid biosynthesis with the production of PE/PC/LPC/LPE (**Figure 4C**). Recent research has also demonstrated the direct bond between ALDOA and PLD2 (29). Therefore, we examined the interaction status between ALDOA and PLD1 or PLD2 through a two-way immunoprecipitation assay (**Figure 4D** and **Supplementary Figure S2**). Our data had the same consistency trend as Kim et al. described with aldolase A binding to the PH domain of PLD2 (37). In contrast, PLD1 did not interact with the ALDOA protein (**Supplementary Figure S2**). In addition to the interaction status, we also screened the protein levels of PLD1 and PLD2. In the ALDOA overexpressing cells or PLD2 knockdown models, the activity of PLD1 is coordinated with ALDOA (**Figure 4E** and **Supplementary Figure S3**). According to previous references, it was suggested that ALDOA interacts with the immobilized 20S proteasome core (38). Meanwhile, we noticed that ubiquitin-related proteins may participate in ALDOA-based proteomics (28). Moreover, we also excluded the influence of epigenetic modification through *in silico* profile analysis (**Supplementary Figure S4**). Therefore, we hypothesized that the ALDOA/PLD2 protein–protein interaction may be through the degradation mechanism of lung cancer cells. We attempted to recruit MG132 to inhibit the proteosome, and our results found that ALDOA did increase the degradation ability in the overexpression model (**Figure 4F**).

### Synergistic Regulation of ALDOA and PLD1 on the Radiation of Lung Cancer Cells

**Figure 5:**
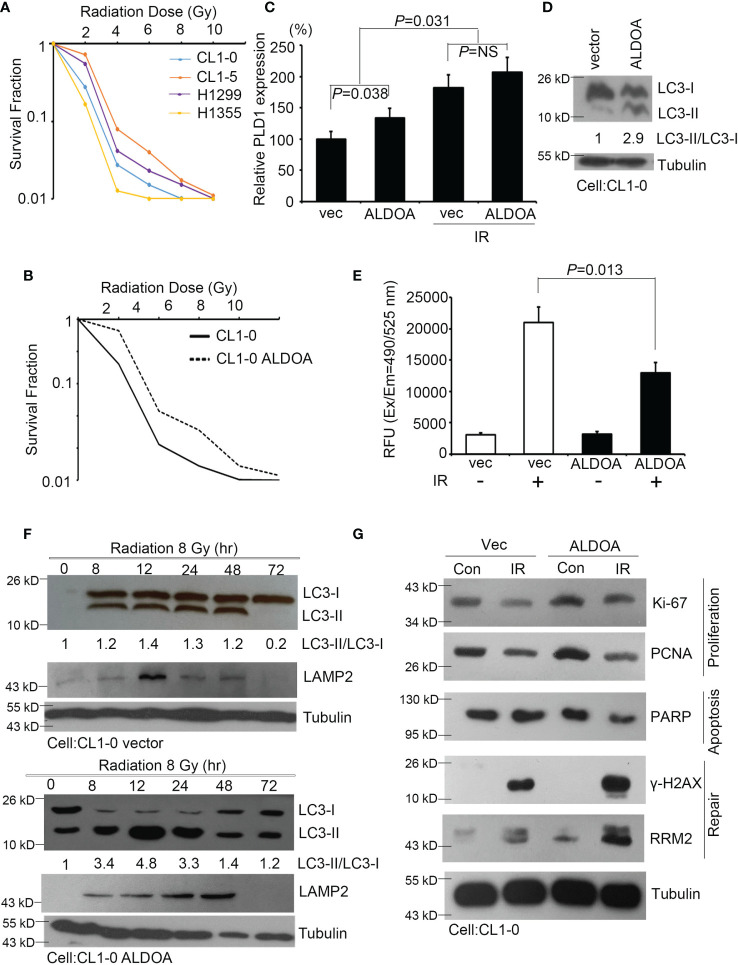
ALDOA increases autophagy and invasion sensitivity after radiation exposure. **(A)** The survival fraction after radiation exposure in various lung cancer cells (CL1-0, CL1-5, H1299, and H1355). Radiation dose: 0~10 Gy. **(B)** The survival fraction after radiation exposure in CL1-0 cells with vector control or ALDOA overexpression. Radiation dose: 0~10 Gy. **(C)** Quantify the expression levels of PLD1 after radiation exposure in CL1-0 cells with vector control or ALDOA overexpression. Radiation dose: 8 Gy **(D)** The LC3-I/II protein level in CL1-0 cells with vector control or ALDOA overexpression. Tubulin serves as an internal control. Radiation dose: 8 Gy **(E)** Quantitation of the activity of caspase-3 in A549 cells after alkylating agent exposure in CL1-0 cells with vector control or ALDOA overexpression. Radiation dose: 8 Gy **(F)** The LC3-I/II and LAMP2 protein level after radiation exposure in a time-dependent manner in CL1-0 cells with vector control or ALDOA overexpression. Tubulin serves as an internal control. Radiation dose: 8 Gy. **(G)** The Ki-67, PCNA, PARP, γ-H2AX, and RRM2 protein level after radiation exposure in CL1-0 cells with vector control or ALDOA overexpression. Tubulin serves as an internal control. Radiation dose: 8 Gy. The data from three independent experiments are presented in **(B–D)** as the means ± SEM. The significance of the difference was analyzed using the nonparametric Mann–Whitney *U* test.

To validate the ability of ALDOA to adapt lung cancer cells exposed to radiation, we screened and determined the survival rate after radiation exposure of various lung cancer cells. As different cells have individual characteristics and heterogenicity, we added more cells and used CL1-0/CL1-5 series cell lines. This series was from the same clinical patients, but the attributes are benign/drug sensitive and malignant/drug resistant, respectively (28, 39). Combined with previous studies on the identification of endogenous ALDOA expression in cancer cell lines, we measured the survival fraction ability of specific lung cancer cells and defined CL1-0 (ALDOA^low^) and H1355 (ALDOA^low^) as radiosensitive cells (28), while CL1-5 (ALDOA^high^) and H1299 (ALDOA^high^) can be regarded as radioresistant cells (**Figure 5A**). We further established an overexpression model in CL1-0 and confirmed that the expression level of ALDOA indeed conferred lung cancer cell viability and radiation response (**Figure 5B** and **Supplementary Figure S5**). Our cell model determined that ALDOA and radiation exposure increase PLD1 expression (**Figure 5C**). In the ALDOA overexpression model, the expression level of PLD1 will increase regardless of irradiation exposure (**Figure 5C**). We further examined the biological effects of radiation in these cells, including autophagy, apoptosis, proliferation, and DNA repair mechanisms. We found that ALDOA overexpression brought a high level of LC3B-II ratio in lung cancer cells (**Figure 5D**). We also examined the overexpression of ALDOA and various phenotype-related markers in radiation. The presence of ALDOA enhanced cell proliferation, DNA repair, and autophagy mechanisms after radiation exposure, and the degree of apoptosis was significantly reduced (**Figures 5E–G**). Merging this evidence, we demonstrated that ALDOA and PLD1 coordinately endow lung cancer cells with resistance to alkylating agents and radiation.

### PLD1 Compensates for Total PLD Enzyme Activity and Phosphatidic Acid Production

**Figure 6:**
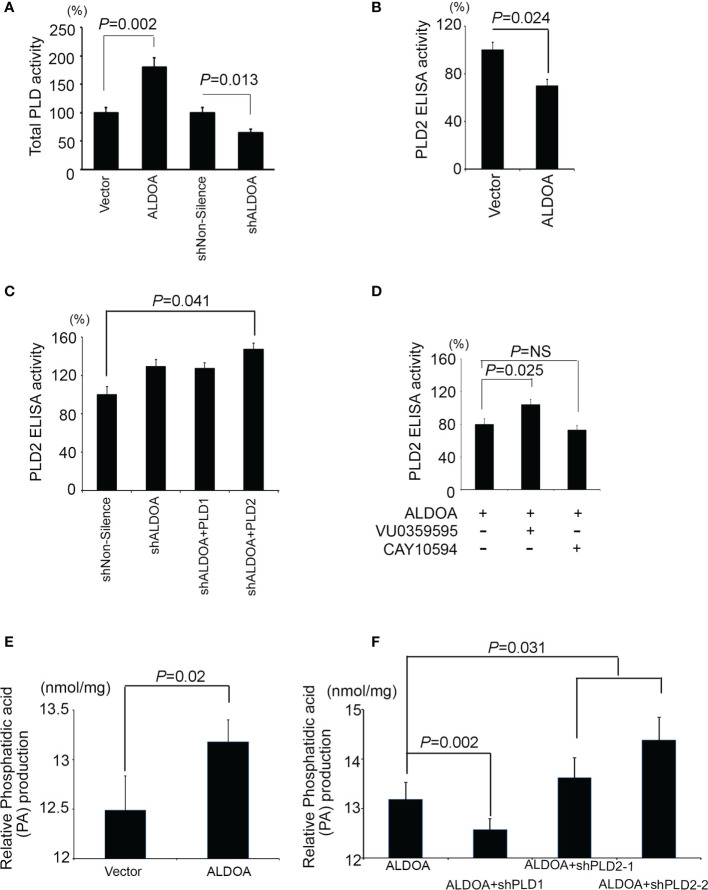
ALDOA interferes with the activity of the PLD2 enzyme but increases the total PLD and phosphatidic acid products. **(A)** Intracellular PLD2 activity in CL1-0 cells with and without ALDOA gene overexpression. **(B)** Intracellular PLD2 activity in ALDOA knockdown CL1-5 cells with and without PLD1 or PLD2 overexpression. **(C)** Total intracellular PLD activity in an ALDOA two-way cell model. **(D)** Intercellular PLD2-specific activity in ALDOA-overexpressing CL1-0 cells treated with pharmaceutical PLD1 inhibitor VU0359595 and PLD2 inhibitor CAY10594, respectively. In this study, 10 μM of VU0359595 and 50 μM of CAY10594. **(E)** Total intracellular phosphatidic acid production in the ALDOA overexpression model. **(F)** Total intracellular phosphatidic acid production in CL1-0 cells overexpressing ALDOA with and without PLD1 or PLD2 gene knockdown by shRNAs. The data from three independent experiments are presented in **(A–F)** as the means ± SEM. The significance of the difference was analyzed using the nonparametric Mann–Whitney *U* test.

According to the aforementioned reference, ALDOA is a component that directly interacts with PLD2 *via* the PH domain and causes PLD2 activity to be inhibited. We suggest that both the total activity of PLD and the production of phosphatidic acid is affected. We detected total PLD enzymatic activity in the ALDOA two-way studies (**Figure 6A**). Interestingly, the total PLD activity is directly regulated by the expression of ALDOA, and the total PLD activity of the ALDOA overexpression group was higher (**Figure 6A**). Subsequently, we further examined the specific expression of PLD2 by ELISA assay. We found that the overexpression of ALDOA can eventually suppress the expression of PLD2, and it can be restored after ALDOA knockdown with or without PLD2 overexpression (**Figures 6B, C**). In addition to the observation results, we also noticed that the position of PLD2 is disturbed when ALDOA is bound, and its content on the membrane is reduced (**Supplementary Figure S6**). We then recruited CAY10594 (specific for PLD2) treatment, which can significantly reduce the ELISA activity of PLD2 in lung cancer cells. It was also determined that VU0359595 (specific for PLD1) did not suppress PLD2 expression (**Figure 6D**). Unfortunately, there is currently no straightforward approach to detect PLD2 enzyme activity alone. In our research, we found that, even if PLD2 is inhibited, the enzyme activity of total PLD still performs, so it is estimated that PLD1 complements or even strengthens the activity of total PLD. Therefore, we will continue to examine whether total PLD activity affects the production of downstream metabolites. Phosphatidic acid (PA) is abundantly expressed in cells with ALDOA overexpression, increasing even more after PLD2 knockdown (**Figures 6E, F**). This may explain why fatty acid reprogramming in cancer can also lead to the activation and reorganization of the cell membrane, which is also consistent with our previous research (40).

### ALDOA-PLD1 Serves as a Prognostic Marker for Lung Cancer Patients

**Figure 7:**
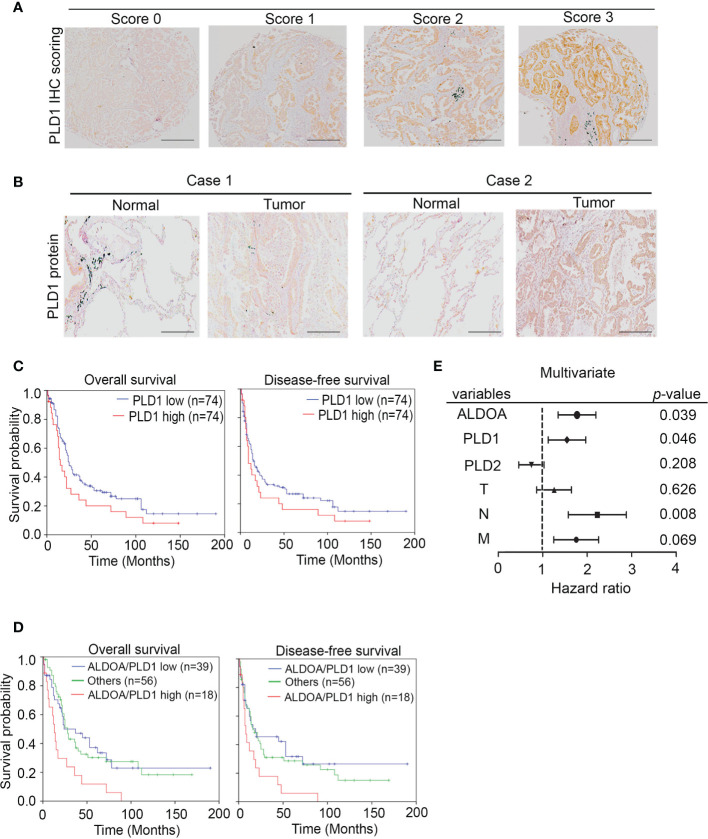
The prognosis value of ALDOA-PLD1 for lung cancer patients. **(A)** Scores (0–3) indicate PLD1 protein levels in representative lung tumor tissues. **(B)** The expression level of the PLD1 protein in tumor tissue compared with the corresponding normal adjacent tissue. **(C)** Kaplan–Meier analysis of PLD1 protein expression at concurrently low or high levels as determined by IHC staining at the endpoint of overall survival probability and disease-free survival probability in lung cancer patients. **(D)** Kaplan–Meier analysis of PLD1 combined with ALDOA protein expression at concurrently low or high levels or others as determined by IHC staining at the endpoint of overall survival probability in lung cancer patients. **(E)** Multivariate analysis of ALDOA/PLD1/PLD2 and clinical parameters in a clinical cohort. The significance of the differences in **(C, D)** was analyzed using the Student’s *t-*test.

To investigate whether the ALDOA/PLD1 axis has clinical significance for cancer patients, we performed the immunohistochemical (IHC) staining of ALDOA, PLD1, and PLD2 with specific antibodies in clinical lung cancer samples to validate these findings (**Figure 7A** and **Supplementary Figure S7**). The IHC results demonstrated that the level of PLD1 protein in patients with lung cancer was elevated. It also predicted poor overall and disease-free survival of patients with lung cancer (**Figures 7B–D**) and was correlated with several clinicopathological factors (**Figure 7E** and **Supplementary Table S1, S2**). In addition, although the reduction in PLD2 protein levels has unfavorable results, it cannot be used as an independent prognostic factor (**Supplementary Figure S6**, **Supplementary Table S3 and S4**). The data indicated that the ALDOA and PLD1 protein levels were positively correlated with the relatively poor overall and disease-free survival rates among lung cancer patients (**Figure 7D**). We further screened for the endogenous expression level between ALDOA and PLD1 in various cancer types through the The Cancer Genome Atlas Program (TCGA) cohorts. We observed that the mRNA level of ALDOA is positively correlated with PLD1, which is consistent with our findings in lung cancer (**Supplementary Figure S8**). Our data confirm that ALDOA can coordinate with PLD1 by regulating the RNA levels of lung cancer patients. The Kaplan–Meier analysis of the lung cancer cohorts includes the TCGA and GEO websites. In addition, this signature can be replicated in multiple probes of the target genes (**Supplementary Figure S9**). Therefore, we claim the ALDOA/PLD1 axis as an independent prognostic factor for lung cancer. These data reveal the crucial role of the ALDOA/PLD1 axis in cancer progression and provide potential therapeutic strategies for cancer treatment in the future.

## Discussion

Locally advanced lung cancer has a high recurrence rate, even after curative treatment with chemotherapy or radiation (41). In one study using stereotactic body radiation for inoperable localized non-small cell lung cancer, the three-year disease-free survival rate was only 48.3% (42). Mechanisms associated with easy relapse after radiation or chemotherapy include tumor microenvironment alterations (43), oxygen concentration (44), and the immune system (45). Oxygen acts as a radiosensitizer (46), but vascular damage caused by radiation can exacerbate tumor hypoxia and lead to HIF-1α dependent consequences (47). Tumor irradiation even induces damage response and leads to the immunogenic cell death (ICD) of cancer cells (48). However, suppressive immune cell enhancement may still limit T-cell activation and checkpoint suppression (49). In addition to the above reasons, metabolic reprogramming is also a key event.

Metabolism reprogramming occurs in various diseases, including cancer. Several metabolites are involved in DNA damage and repair (50). In particular, lipid metabolism is considered one of the specific reprogramming events that relates antioxidants and DNA double-strand breaks through lipid peroxidation (51). Moreover, cancer cells attacked by radiation and alkylating agents may cause similar reactions. In lung cancer, glucose, lipid, and mitochondria metabolism dysfunction have been mentioned (52). Some agents targeting cancer metabolism have been developed in recent years, such as 3-BP, sodium oxamate, orlistat, etc. (52). Their derived preclinical trial compounds include metformin, gossypol, and enasidenib, etc. (52). In addition, metabolism is involved in DNA repair, which is crucial for its synthetically anti-cancer effect. For example, poly(ADP-ribose) polymerase (PARP) inhibitors can be used as sensitizers to enhance the cytotoxicity of ionizing radiation and alkylating agents through DNA repair mechanisms (53). Phospholipase D also participates and plays an important role in these events. Our research not only proves that the regulation of phospholipase D depends on PLD1 in lung cancer, but it also shows that ALDOA is required as a gatekeeper for glycolytic conversion and intermediate production.

Mammalian cells encode two isoforms of PLD, PLD1, and PLD2 (54). Both are responsible for PC hydrolysis and PA production (55). PLD1 has been shown to interact with vascular endothelial cells and promote the release of vascular endothelial growth factor (VEGF) to the tumor microenvironment (56, 57). In contrast, PLD2 phosphorylates Janus Kinase3 (JAK3) has been reported to promote cell invasion in breast cancer (58, 59). As previously reported, PLD1 and PLD2 rely on different signals and their corresponding phenotypes in multiple cancers (15, 60). Therefore, several potent PLD inhibitors, including the neuropsychiatric drug halopemide (61, 62), have been developed for anti-cancer treatments. Inhibitors selective for PLD1 or PLD2 specifically suppress gene expression or enzyme activity (63, 64). Kang et al. proposed that PLD1 and PLD2 enzymatic activities are essential. Still, it is noted that only PLD1 is selectively induced through a positive feedback loop to prolong and amplify the response to the signal (65, 66). PLD is involved in numerous cellular functions, such as exocytosis, vesicle trafficking, the cell cytoskeleton, and proliferation (67). PLD members also control survival and migration in tumor cell lines (22). However, the structure, function, and intracellular localization of PLD members vary in different cell types or tissues (54, 68–70). Wild-type PLD2 was found to be increased in EL4 lymphoma metastasis *in vivo*, but an inactive form of PLD2 was associated with fewer liver metastases than those of control cells (71). In addition, PLD1 plays a role in melanoma growth and metastasis *in vivo*; a significant reduction in tumor metastasis was observed in the wild-type or PLD1 knockout mice following CAY10594 inhibitor treatment (72, 73). Several reports have focused on the PLD1/PLD2 balance and switch in different cancer types (74); however, there are few reports on lung cancer. Therefore, we studied the role of PLD1/PLD2 in lung cancer and assessed whether enzyme activity and protein function are involved in lung cancer progression. We demonstrated that PLD1 could be activated to restore PLD activity and PA production following PLD2 loss of function through direct interaction with aldolase A. Using knockdown clones and specific inhibitors, we demonstrated that PLD1 plays a significant role in maintaining PA production and enhancing downstream signaling in lung cancer, whereas PLD2 does not. We currently speculate that PLD1 can dominate lung cancer metastasis and supplement the production of PA after the function of PLD2 is attenuated or even more, but this requires further research.

Furthermore, a high expression of aldolase A combined with PLD1 synergistically indicated a poor survival rate in lung cancer patients, consistent with the cell model. Since both radiation and alkylating agents are important as adjuvant therapy for preventing recurrence and metastases in locally advanced non-small cell lung cancer, our results may provide new insights into improving clinical outcomes by targeting the novel ALDOA/PLD1 axis. In addition, previous studies have also shown that PLD2 had carcinogenic effects and correlated with a poor prognosis (75, 76). Therefore, a more precise classification for specific cancer types that depend on ALDOA and downstream signaling may be an important direction for further research.

## Conclusions

This study provided evidence that ALDOA directly binds to PLD2 and suppresses its enzymatic activity, while PLD1 compensates for and enhances proliferation, repair, and anti-apoptotic capabilities. At the same time, along with alkylating agents or radiation exposure, ALDOA and PLD1 jointly support various aggressive cancer phenotypes and the metabolic reprogramming of lung cancer cells. Most importantly, ALDOA and PLD1 have predictive value for the survival of lung cancer patients. Thus, ALDOA-PLD1 should be monitored in the future, and targeting the ALDOA/PLD1 axis should be developed as a therapy for lung cancer patients.

## Data Availability Statement

The original contributions presented in the study are included in the article/**Supplementary Material**. Further inquiries can be directed to the corresponding author.

## Ethics Statement

The study was performed with the approval of the institutional review board and with permission from the ethics committee of the institution involved (KMUH-IRB-2011-0286). The patients/participants provided their written informed consent to participate in this study.

## Author Contributions

Conception and design: Y-CC, M-HsC, and MH. Development of methodology: Y-CC and M-HsC. Acquisition of data (provided animals, acquired and managed patients, provided facilities, etc.): Y-CC, M-HsC, M-HuC, and Y-JL. Analysis and interpretation of data (e.g., statistical analysis, biostatistics, computational analysis): Y-CC, M-HsC, Y-JL, and MH. Writing, review, and/or revision of the manuscript: Y-CC, M-HsC, and MH. Administrative, technical, or material support (i.e., reporting or organizing data, constructing databases): M-HsC, M-HuC, and MH. Study supervision: MH. All authors contributed to the article and approved the submitted version.

## Funding

This study was supported by Academia Sinica and Ministry of Science and Technology: (AS-SUMMIT-108), (AS-SUMMIT-109), (ASKPQ-109-BioMed), (MOST-108-3114-Y-001-002), and (MOST 107-2320-B-001 -016 -MY3) to MH. This study was also supported by Ministry of Science and Technology (MOST-110-2320-B-010-008-MY2), Yen Tjing Ling Medical Foundation (CI-111-9) and Veterans General Hospitals and University System of Taiwan Joint Research Program (VGHUST111-G3-3-2 ) to Y-CC.

## Conflict of Interest

The authors declare that the research was conducted in the absence of any commercial or financial relationships that could be construed as a potential conflict of interest.

## Publisher’s Note

All claims expressed in this article are solely those of the authors and do not necessarily represent those of their affiliated organizations, or those of the publisher, the editors and the reviewers. Any product that may be evaluated in this article, or claim that may be made by its manufacturer, is not guaranteed or endorsed by the publisher.
