# Supplementary material for: Aldolase A and Phospholipase D1 Synergistically Resist Alkylating Agents and Radiation in Lung Cancer
Source: Front Oncol. 2022 Jan 21;11:811635. doi: 10.3389/fonc.2021.811635 (PMC8813753; doi:10.3389/fonc.2021.811635)
Supplement: Supplementary file 1 [file DataSheet_1.docx]

**Supplementary information**

**Aldolase A and Phospholipase D1 synergistically resist alkylating agents and radiation in lung cancer**

Yu-Chan Chang^1^, Peter Mu-Hsin Chang^2,3,4^, Chien-Hsiu Li^5^, Ming-Hsien Chan^5^, Yi-Jang Lee^1^, Ming-Huang Chen^3,6^, Michael Hsiao^5,7*^

1. Department of Biomedical Imaging and Radiological Science, National Yang Ming Chiao Tung University, Taipei, Taiwan
2. Department of Oncology, Taipei Veterans General Hospital, Taipei, Taiwan
3. Faculty of Medicine, National Yang Ming Chiao Tung University, Taipei, Taiwan
4. Institute of Biopharmaceutical Sciences, National Yang Ming Chiao Tung University, Taipei, Taiwan
5. Genomics Research Center, Academia Sinica, Taipei, Taiwan
6. Center of Immuno-Oncology, Department of Oncology, Taipei Veterans General Hospital, Taipei, Taiwan
7. Department of Biochemistry, College of Medicine, Kaohsiung Medical University, Kaohsiung, Taiwan

*To whom correspondence should be addressed:

Michael Hsiao, Genomics Research Center, Academia Sinica, 128 Academia Rd., Sec. 2, Nankang-Dist., Taipei, Taiwan. Tel: +886-2-2787-1243, Fax: +886-2-2789-9931, E-mail: [mhsiao@gate.sinica.edu.tw](mailto:mhsiao@gate.sinica.edu.tw)

**Supplementary table and table legends**

**Table S1.** Clinical relevance of PLD1 expression in lung cancer. **p* value<0.05 was considered statistically significant (Student’s *t*-test for continuous variables and Pearson’s chi-square test for variables). SD represents the standard deviation. ^#^ The tumor stage, tumor status, lymph node, and distal metastasis status were classified according to the international system for staging lung cancer.

|  | PLD1 expression, n (%) | | | | | |
| --- | --- | --- | --- | --- | --- | --- |
| Characteristics | | n | Low  (n =122) | High  (n =26) | *P* value |  |
| Age | |  |  |  |  |  |
| ＜65y  ≧65y | | 78  70 | 65(83.3)  57(81.4) | 13(16.7)  13(18.6) | 0.761 |  |
| Sex | |  |  |  |  |  |
| Male | | 85 | 74(87.1) | 11(12.9) | 0.086 |  |
| Female | | 63 | 48(76.2) | 15(23.8) |  |  |
| Smoking status | |  |  |  |  |  |
| No | | 87 | 72(82.8) | 15(17.2) | 0.901 |  |
| Yes | | 61 | 50(82.0) | 11(18.0) |  |  |
| Histological type | |  |  |  |  |  |
| Adenocarcinoma | | 97 | 73(75.3) | 24(24.7) | 0.006 |  |
| Squamous carcinoma | | 41 | 39(95.1) | 2(4.9) |  |  |
| Large cell carcinoma | | 10 | 10(100.0) | 0(0.0) |  |  |
| Stage^#^ | |  |  |  |  |  |
| I+ II | | 58 | 53(91.4) | 5(8.6) | 0.022^*^ |  |
| III+ IV | | 90 | 69(76.7) | 21(23.3) |  |  |
| Tumor status | |  |  |  |  |  |
| T1+ T2 | | 105 | 87(82.9) | 18(17.1) | 0.832 |  |
| T3+ T4 | | 43 | 35(81.4) | 8(18.6) |  |  |
| Lymph node status | |  |  |  |  |  |
| N0 | | 51 | 46(90.2) | 5(9.8) | 0.072 |  |
| N1-3 | | 97 | 76(78.4) | 21(21.6) |  |  |
| Distal metastasis status | |  |  |  |  |  |
| M0 | | 102 | 86(84.3) | 16(15.7) | 0.370 |  |
| M1 | | 46 | 36(78.3) | 10(21.7) |  |  |
| Recurrence status | |  |  |  |  |  |
| No | | 36 | 33(91.7) | 3(8.3) | 0.094 |  |
| Yes | | 112 | 89(79.5) | 23(20.5) |  |  |
|  | |  |  |  |  |  |

**Table S2.** Univariate and multivariate analyses for PLD1 expression in lung cancer. **p* value<0.001 was considered significant.

| Cox univariate analysis (OS) | | |  |  |  |
| --- | --- | --- | --- | --- | --- |
| Variables | | Comparison | HR (95% CI) | | *P*-value |
| T |  | T3-T4 vs. T1-T2 | 1.683 (1.127-2.513) | | 0.0110 |
| N |  | N1-N3 vs. N0 | 2.871 (1.906-4.324) | | <0.0001^*^ |
| M |  | M1 vs. M0 | 2.693 (1.809-4.007) | | <0.0001^*^ |
| PLD1 |  | High vs. Low | 1.741 (1.495-2.110) | | 0.0147 |
| Cox multivariate analysis (OS) | | |  |  |  |
| Variables | | Comparison | HR (95% CI) | | *P*-value |
| T |  | T3-T4 vs. T1-T2 | 0.996 (0.640-1.550) | | 0.985 |
| N |  | N1-N3 vs. N0 | 2.737 (1.757-4.265) | | <0.0001 |
| M |  | M1 vs. M0 | 2.212 (1.452-3.368) | | <0.0001 |
| PLD1 |  | High vs. Low | 1.670 (1.440-2.021) | | 0.0062 |
| Cox univariate analysis (DFS) | | |  |  |  |
| Variables | | Comparison | HR (95% CI) | | *P*-value |
| T |  | T3-T4 vs. T1-T2 | 1.720 (1.156-2.558) | | 0.007 |
| N |  | N1-N3 vs. N0 | 3.142 (2.089-4.728) | | <0.0001^*^ |
| M |  | M1 vs. M0 | 2.260 (1.528-3.343) | | <0.0001^*^ |
| PLD1 |  | High vs. Low | 1.801 (1.537-2.193) | | 0.027 |
| Cox multivariate analysis (DFS) | | |  |  |  |
| Variables | | Comparison | HR (95% CI) | | *P*-value |
| T |  | T3-T4 vs. T1-T2 | 1.057 (0.669-1.670) | | 0.813 |
| N |  | N1-N3 vs. N0 | 2.812 (1.830-4.322) | | <0.0001 |
| M |  | M1 vs. M0 | 1.805 (1.169-2.789) | | 0.008 |
| PLD1 |  | High vs. Low | 1.759 (1.504-2.144) | | 0.188 |

**Table S3.** Clinical relevance of PLD2 expression in lung cancer. **p* value<0.05 was considered statistically significant (Student’s *t*-test for continuous variables and Pearson’s chi-square test for variables). SD represents the standard deviation. ^#^ The tumor stage, tumor status, lymph node, and distal metastasis status were classified according to the international system for staging lung cancer.

|  | PLD2 expression, n (%) | | | | | |
| --- | --- | --- | --- | --- | --- | --- |
| Characteristics | | n | High  (n =89) | Low  (n =59) | *P* value |  |
| Age | |  |  |  |  |  |
| ＜65y  ≧65y | | 78  70 | 50(64.1)  39(55.7) | 28(35.9)  31(44.3) | 0.298 |  |
| Sex | |  |  |  |  |  |
| Male | | 85 | 50(58.8) | 35(41.2) | 0.705 |  |
| Female | | 63 | 39(61.9) | 24(38.1) |  |  |
| Smoking status | |  |  |  |  |  |
| No | | 87 | 55(63.2) | 32(36.8) | 0.360 |  |
| Yes | | 61 | 34(55.7) | 27(44.3) |  |  |
| Histological type | |  |  |  |  |  |
| Adenocarcinoma | | 97 | 71(73.2) | 26(26.8) | <0.001 |  |
| Squamous carcinoma | | 41 | 16(39.0) | 25(61.0) |  |  |
| Large cell carcinoma | | 10 | 2(20.0) | 8(80.0) |  |  |
| Stage^#^ | |  |  |  |  |  |
| I+ II | | 58 | 36(62.1) | 22(37.9) | 0.700 |  |
| III+ IV | | 90 | 53(58.9) | 37(41.1) |  |  |
| Tumor status | |  |  |  |  |  |
| T1+ T2 | | 105 | 66(62.9) | 39(37.1) | 0.291 |  |
| T3+ T4 | | 43 | 23(53.5) | 20(46.5) |  |  |
| Lymph node status | |  |  |  |  |  |
| N0 | | 51 | 31(60.8) | 20(39.2) | 0.907 |  |
| N1-3 | | 97 | 58(59.8) | 39(40.2) |  |  |
| Distal metastasis status | |  |  |  |  |  |
| M0 | | 102 | 68(66.7) | 34(33.3) | 0.016^*^ |  |
| M1 | | 46 | 21(45.7) | 25(54.3) |  |  |
| Recurrence status | |  |  |  |  |  |
| No | | 36 | 22(61.1) | 14(38.9) | 0.891 |  |
| Yes | | 112 | 67(59.8) | 45(40.2) |  |  |
|  | |  |  |  |  |  |

**Table S4.** Univariate and multivariate analyses for PLD2 expression in lung cancer. **p* value<0.001 was considered significant.

| Cox univariate analysis (OS) | | |  |  |  |
| --- | --- | --- | --- | --- | --- |
| Variables | | Comparison | HR (95% CI) | | *P*-value |
| T |  | T3-T4 vs. T1-T2 | 1.876 (1.208-2.913) | | 0.005 |
| N |  | N1-N3 vs. N0 | 2.818 (1.801-4.411) | | <0.0001^*^ |
| M |  | M1 vs. M0 | 2.597 (1.684-4.004) | | <0.0001^*^ |
| PLD2 |  | High vs. Low | 0.999 (0.997-1.001) | | 0.493 |
| Cox multivariate analysis (OS) | | |  |  |  |
| Variables | | Comparison | HR (95% CI) | | *P*-value |
| T |  | T3-T4 vs. T1-T2 | 1.152 (0.699-1.897) | | 0.580 |
| N |  | N1-N3 vs. N0 | 2.391 (1.465-3.903) | | <0.0001 |
| M |  | M1 vs. M0 | 2.033 (1.274-3.245) | | 0.003 |
| PLD2 |  | High vs. Low | 0.999 (0.997-1.001) | | 0.347 |
| Cox univariate analysis (DFS) | | |  |  |  |
| Variables | | Comparison | HR (95% CI) | | *P*-value |
| T |  | T3-T4 vs. T1-T2 | 1.293 (0.841-1.988) | | 0.241 |
| N |  | N1-N3 vs. N0 | 1.194 (0.787-1.811) | | 0.405 |
| M |  | M1 vs. M0 | 1.196 (0.793-1.806) | | 0.393 |
| PLD2 |  | High vs. Low | 0.766 (0.487-1.207) | | 0.250 |
| Cox multivariate analysis (DFS) | | |  |  |  |
| Variables | | Comparison | HR (95% CI) | | *P*-value |
| T |  | T3-T4 vs. T1-T2 | 1.161 (0.723-1.864) | | 0.538 |
| N |  | N1-N3 vs. N0 | 1.168 (0.759-1.799) | | 0.480 |
| M |  | M1 vs. M0 | 1.109 (0.708-1.736) | | 0.652 |
| PLD2 |  | High vs. Low | 0.814 (0.506-1.310) | | 0.396 |

**Supplementary Figure legends**


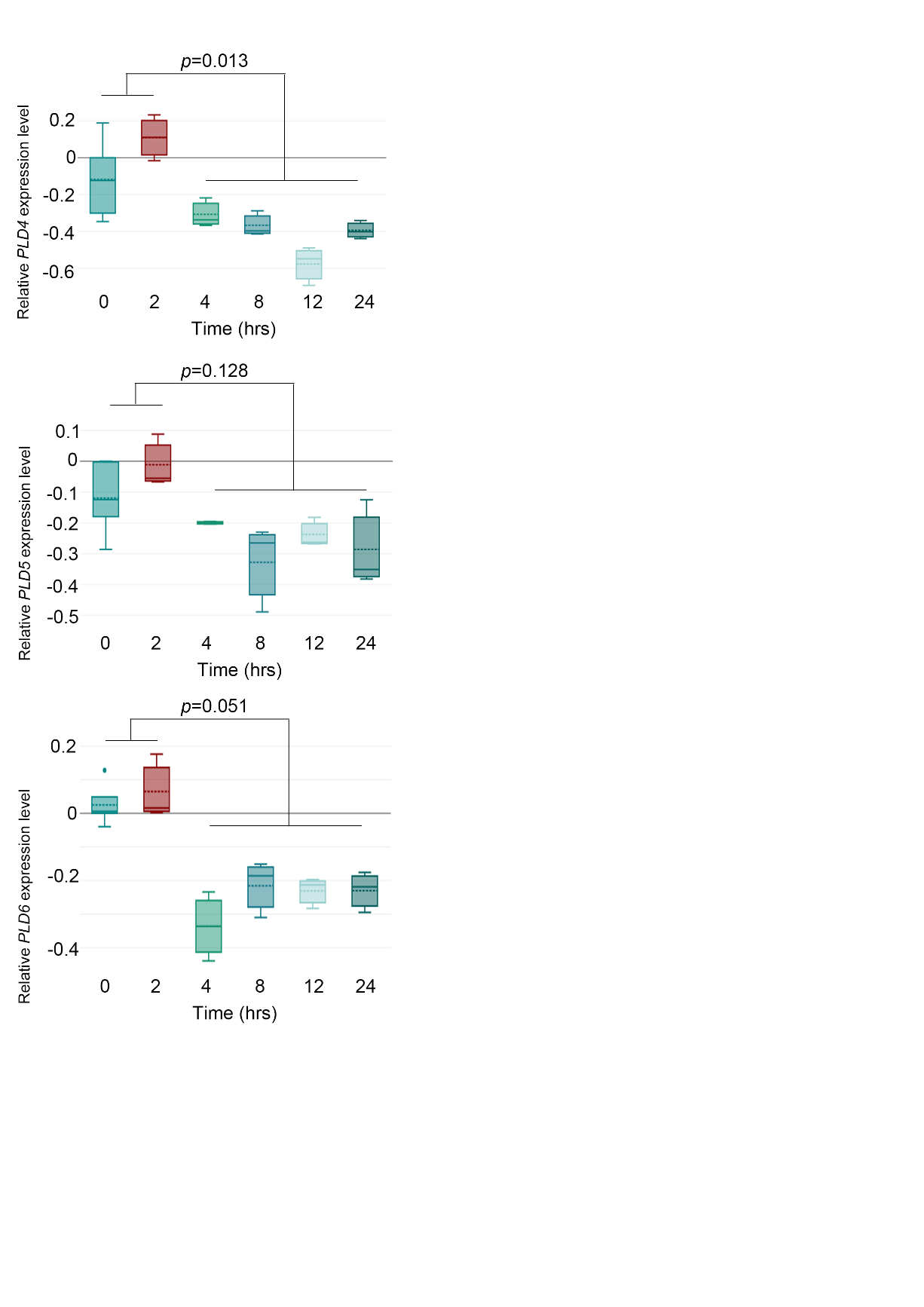


**Figure S1. The radiation response of lung cancer cells and the expression level of the PLD family.** Quantify the expression levels of PLD4 (upper), PLD5 (middle), and PLD6 (lower) at each time point (0, 2, 4, 8, 12, and 24 hours) after radiation exposure in the GSE20549 profile. The significance of the difference was analyzed using the nonparametric Mann-Whitney *U*-test.

**
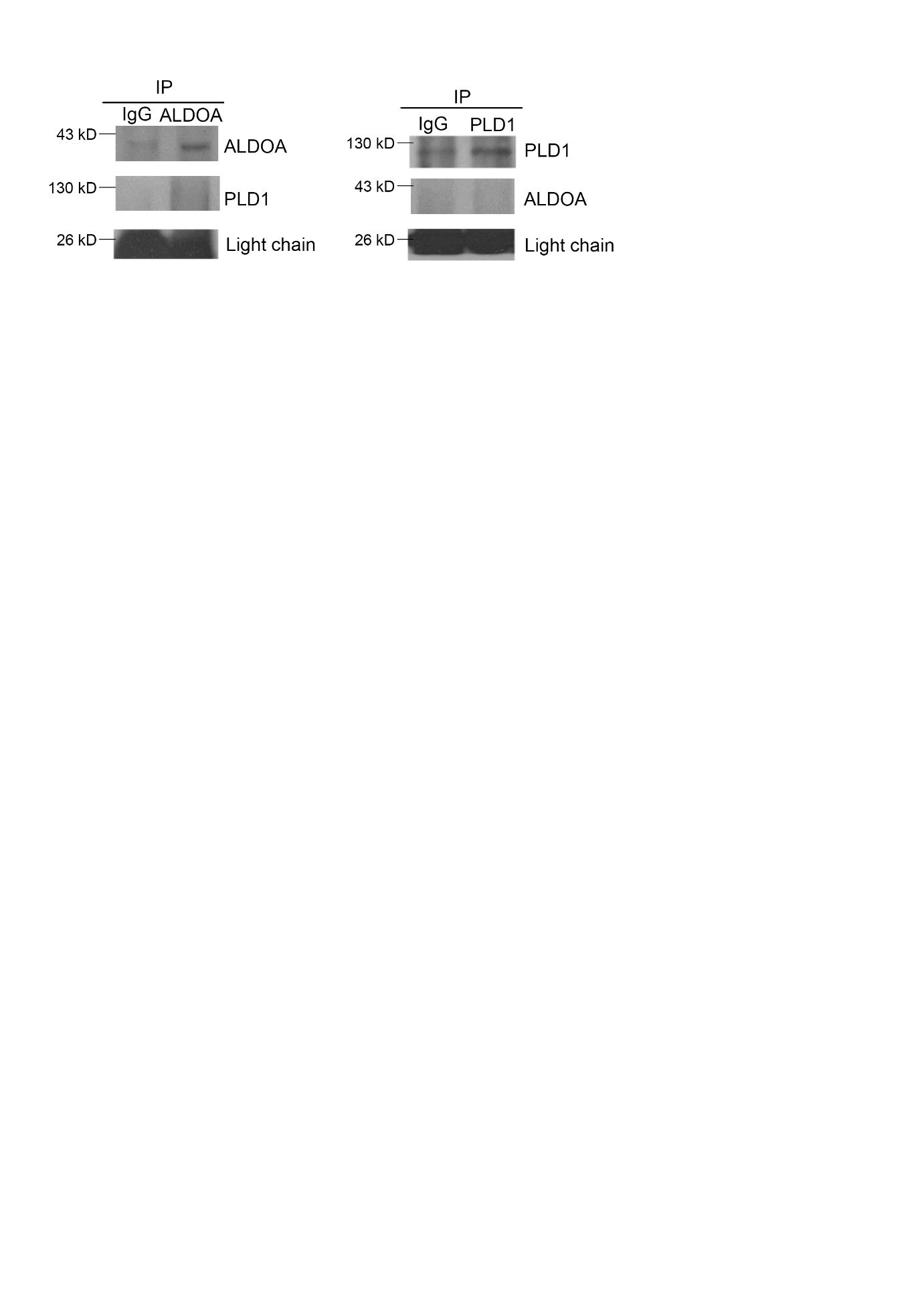
**

**Figure S2.** Two-way model of immunoprecipitation using ALDOA and PLD1 antibodies in CL1-0 cells with or without forced expression of an exogenous ALDOA gene. IgG served as the negative control.


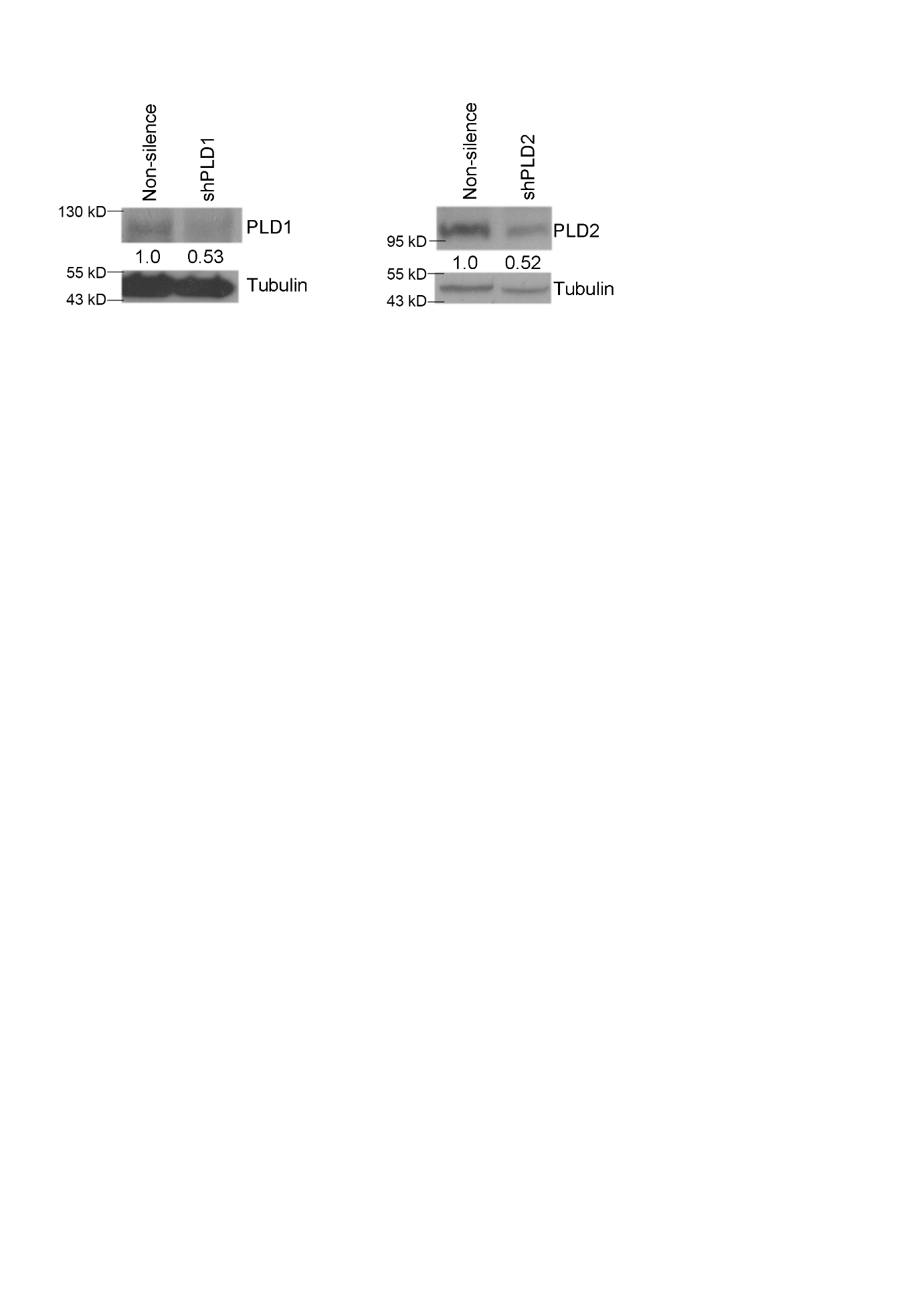


**Figure S3. PLD1 and PLD2 knockdown models were established in the ALDOA overexpression cell.** Western blot analysis of PLD1 and tubulin protein expression in ALDOA-overexpressing CL1-0 cells with the forced expression of non-silencing (NS), PLD1 (left panel), or PLD2 (right panel) shRNAs. Tubulin was used as an internal control for protein loading.

**
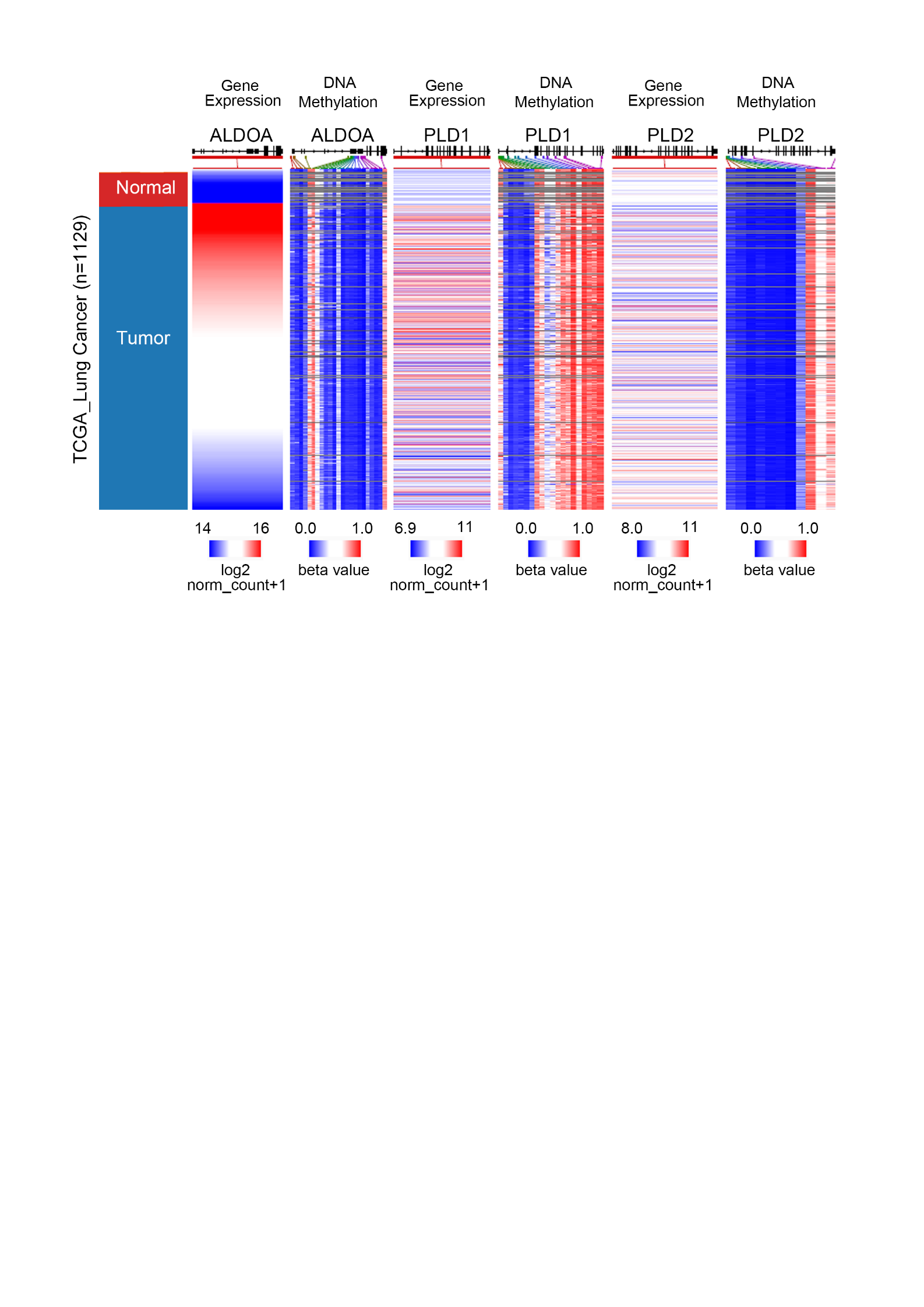
**

**Figure S4. DNA methylation and gene expression of ALDOA, PLD1, and PLD2 in TCGA lung cancer patients.** The data was obtained from the Xena server, the gene expression was obtained by RNA-seq (IlluminaHiSeq), and the methylation status was obtained by Methylation450k events. A total of 1129 clinical cases.

**
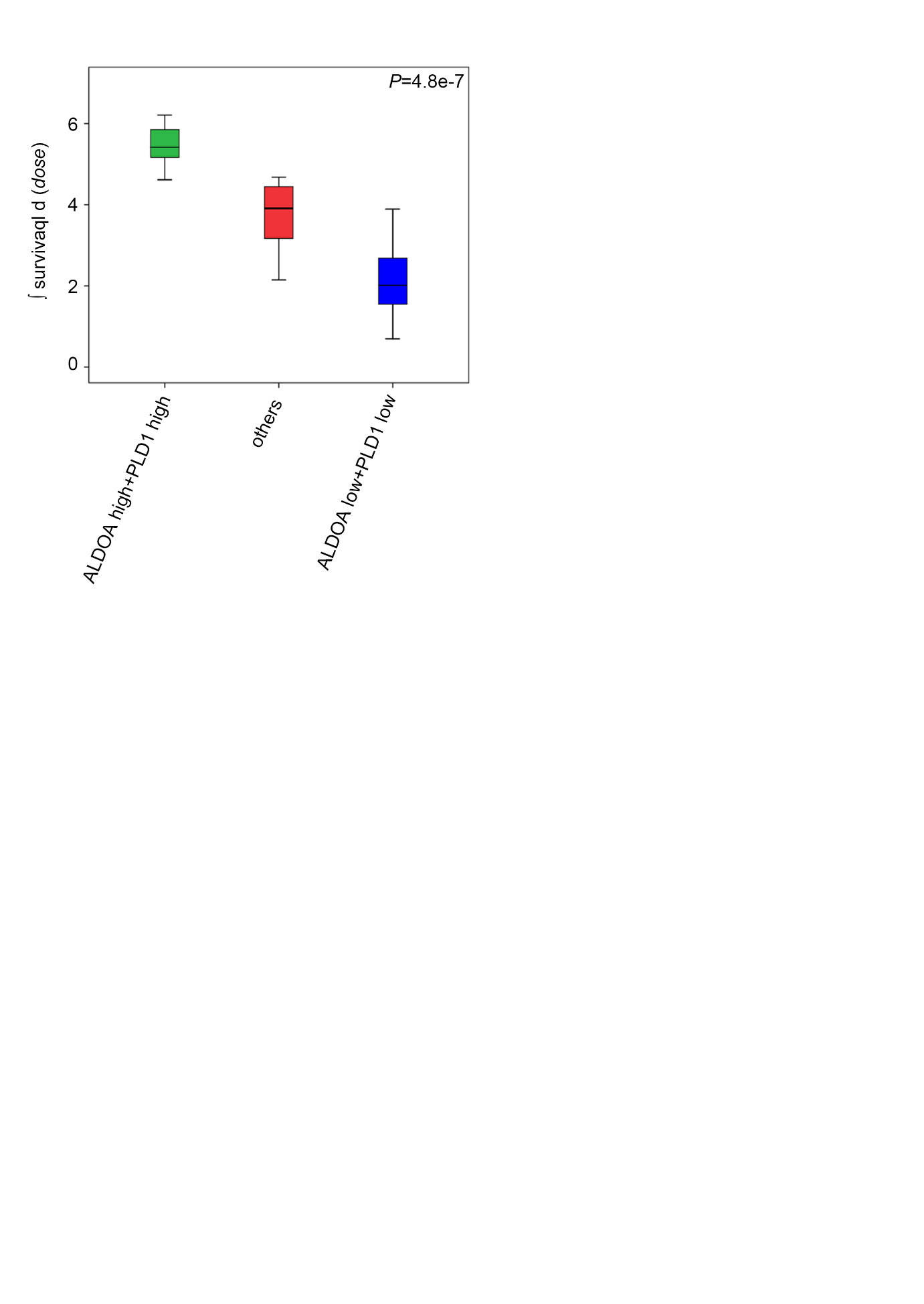
**

**Figure S5. ALDOA/PLD1 can be used as an indicator of lung cancer cell radiation response.** The survival rates of various lung cancer cells were divided into three groups based on the expression level of ALDOA and PLD1. The significance of the difference was analyzed using the nonparametric Mann-Whitney *U*-test.


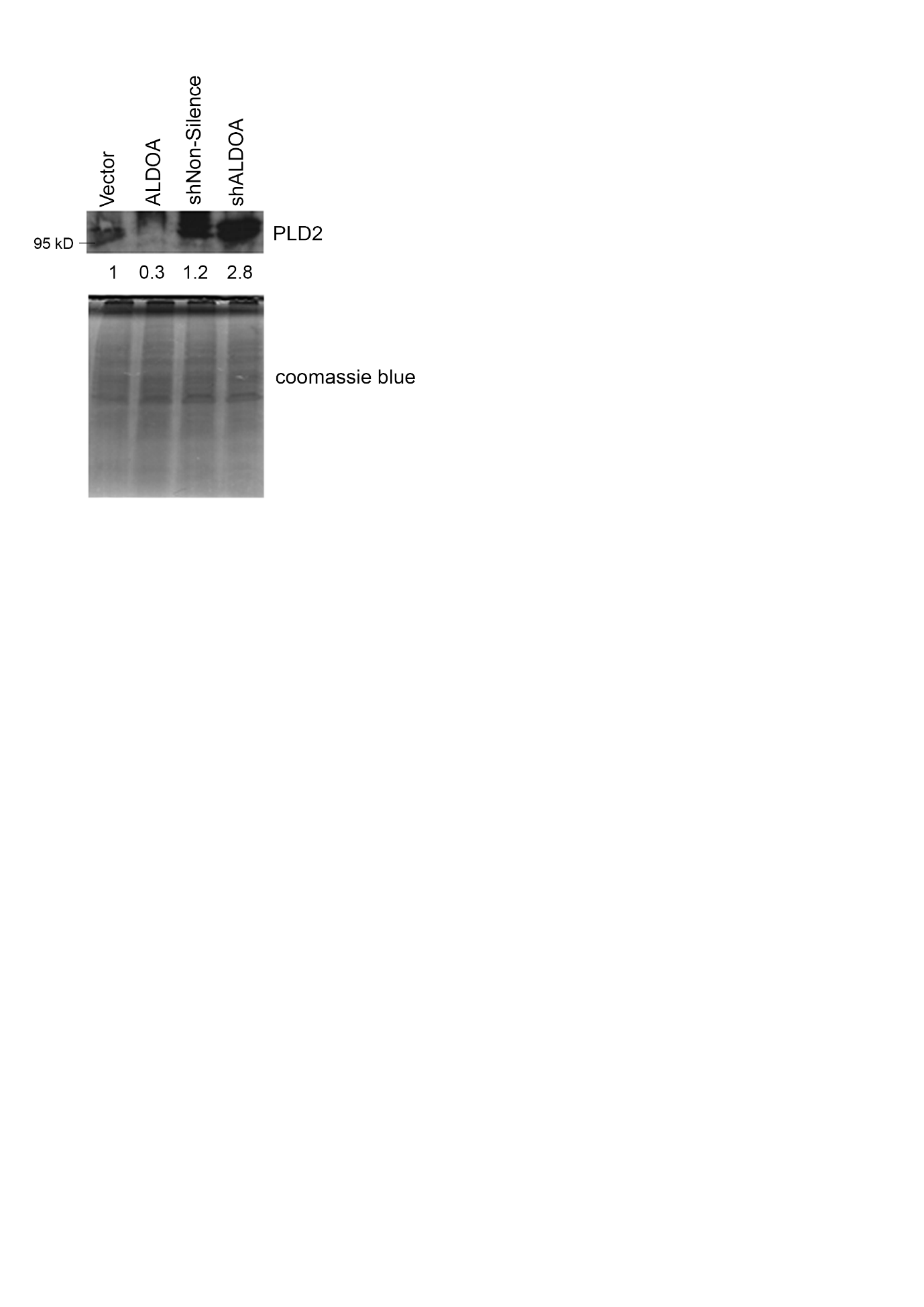


**Figure S6. Western blot analysis of PLD2 protein levels in the membrane protein fraction of cells obtained from the ALDOA two-way model.** Coomassie blue stain served as an internal control for protein loading.

**
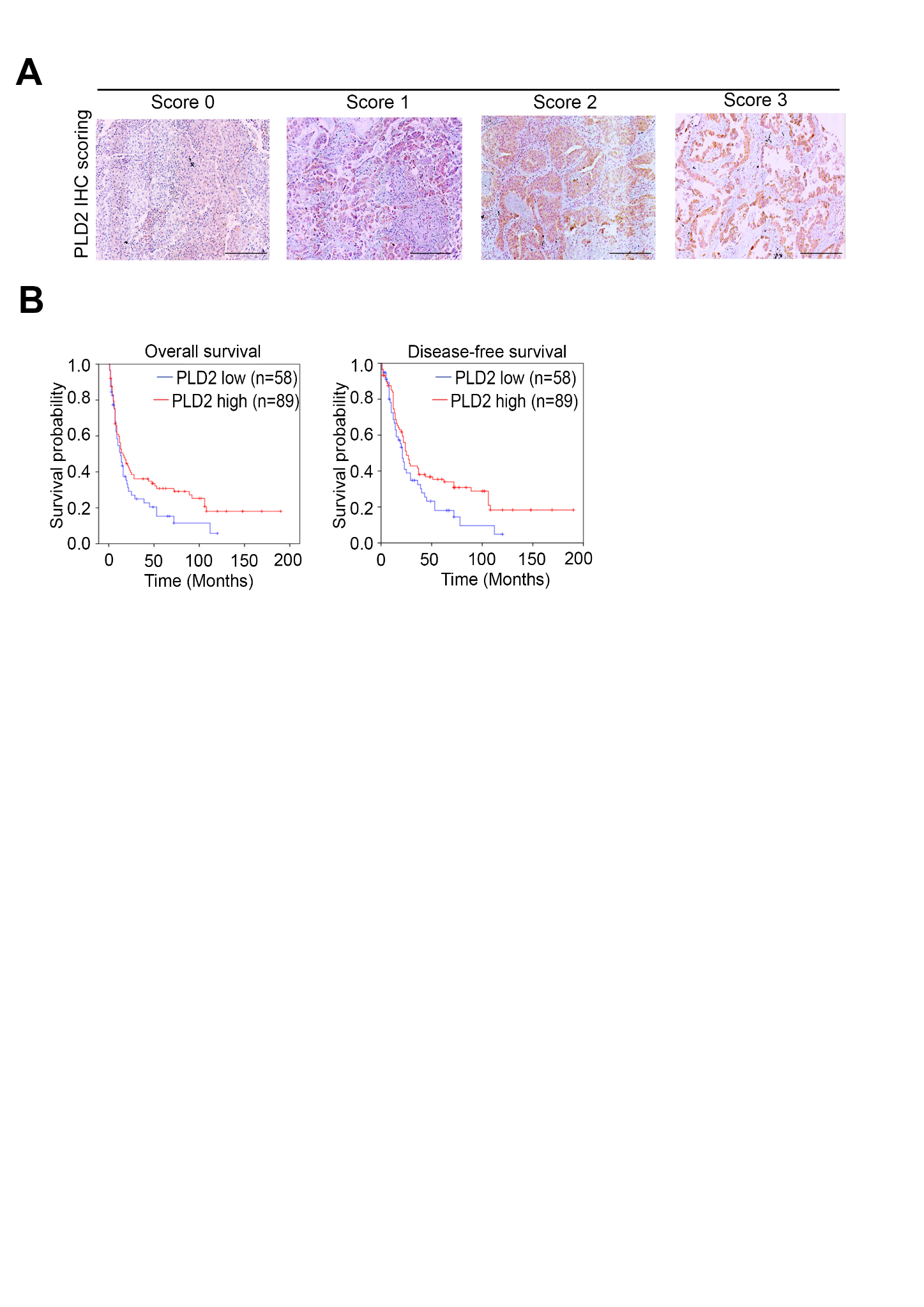
**

**Figure S7.** **PLD2 protein profiles in lung cancer patients.** (**A)** Scores (0-3) indicate PLD2 protein levels in representative lung tumor tissues. (**B)** Kaplan-Meier analysis of PLD2 protein expression at concurrently low or high levels as determined by IHC staining at the endpoint of overall survival probability and disease-free survival probability in lung cancer patients. The significance of the differences in **B** was analyzed using the Student *t* test.


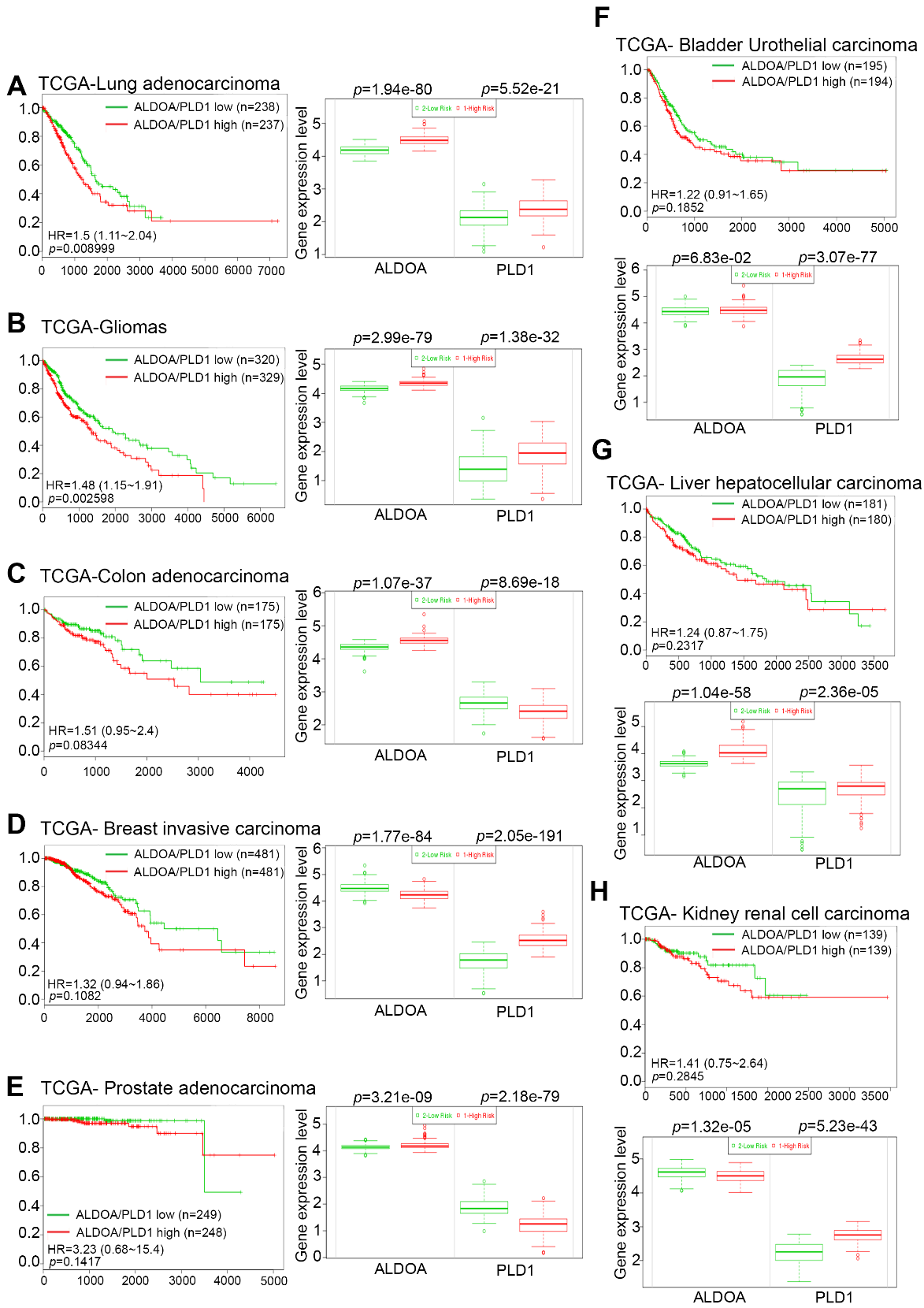


**Figure S8.** ***In silico* prediction, the ALDOA-PLD1 axis of clinical function.** Kaplan-Meier analysis of PLD1 combined with ALDOA gene expression as identified in the (A) TCGA_LUAD cohort, (B) TCGA_Gliomas, (C) TCGA_COAD, (D) TCGA_BRCA, (E) TCGA_PRAD, (F) TCGA_BLCA, (G) TCGA_LIHC, and (H) TCGA_KIRC from the Survexpress website with the endpoint of overall survival, respectively.

**
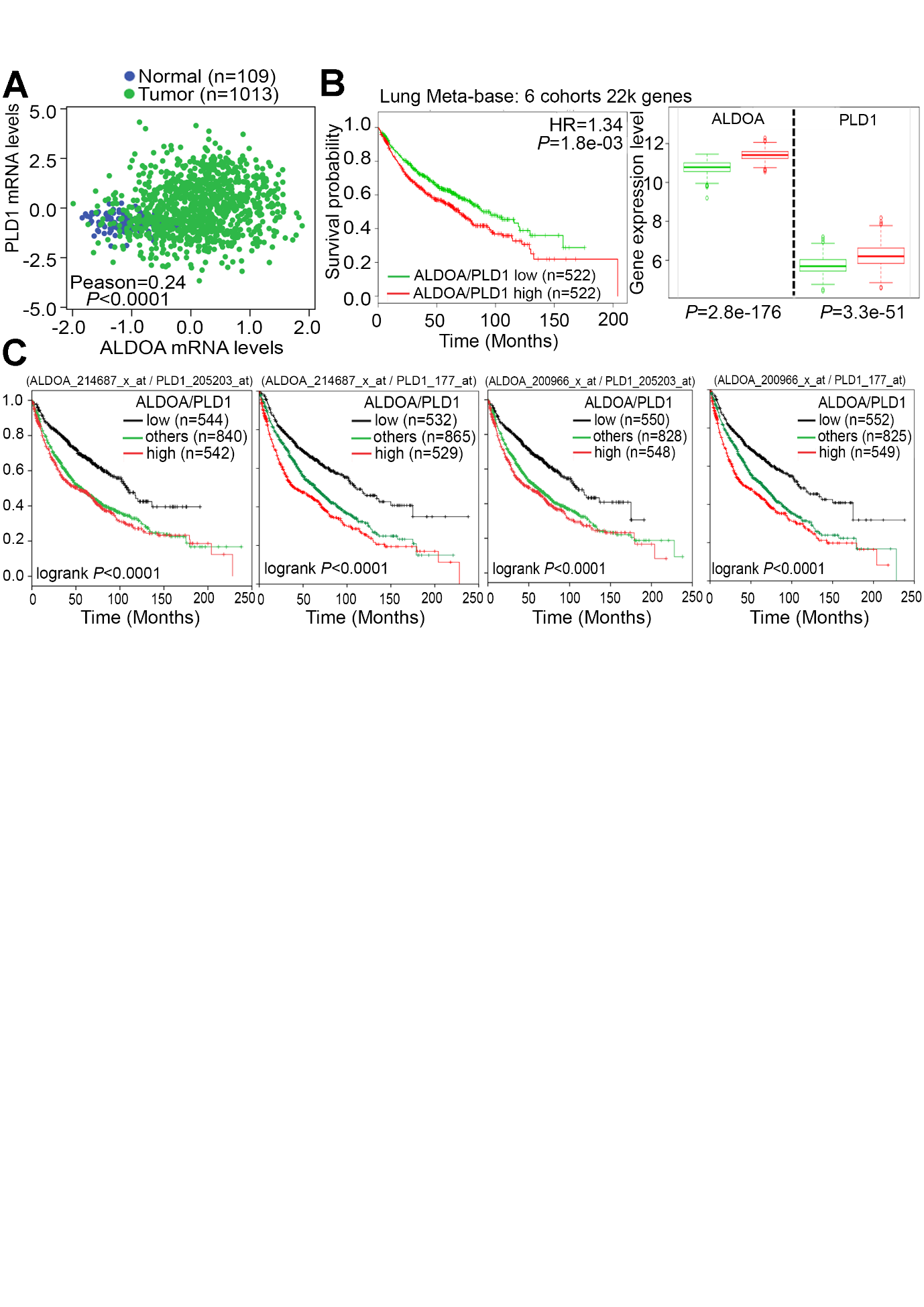
**

**Figure S9. *In silico* prediction, the ALDOA-PLD1 axis of clinical function. (A)** The correlation between ALDOA and PLD1 gene expression from TCGA RNA sequencing results in non-tumor and tumor tissues derived from lung cancer patients. The significance of the correlation was analyzed by Pearson’s method. (**B)** Kaplan-Meier analysis of PLD1 combined with ALDOA gene expression as identified in the meta-base cohort (Lung Meta-base: 6 cohorts 22k genes, n=1044) from the Survexpress website with the endpoint of overall survival. (**C)** Kaplan-Meier analysis of ALDOA (probe ID= 214687_x_at and 200966_x_at) combined with PLD1 (probe ID=205203_at and 177_at) gene expression at concurrently low or high levels or others at the endpoint of overall survival probability from the Kaplan-Meier Plotter database in lung cancer patients (n=192).
